# Intraspecific competition reduces niche width in experimental populations

**DOI:** 10.1002/ece3.1254

**Published:** 2014-09-30

**Authors:** Christine E Parent, Deepa Agashe, Daniel I Bolnick

**Affiliations:** 1 Section of Integrative Biology, University of Texas at Austin1 University Station C0930, Austin, Texas, 78712; 2 Department of Organismic and Evolutionary Biology, Harvard University16 Divinity Avenue, Cambridge, Massachusetts, 02138; 3 National Centre for Biological Sciences, Tata Institute of Fundamental ResearchGKVK, Bellary Road, Bangalore, 560065, India; 4 Howard Hughes Medical Institute1 University Station C0930, Austin, Texas, 78712

**Keywords:** density-dependence, ideal free distribution, maladaptation, niche expansion, optimality, *Tribolium castaneum*

## Abstract

Intraspecific competition is believed to drive niche expansion, because otherwise suboptimal resources can provide a refuge from competition for preferred resources. Competitive niche expansion is well supported by empirical observations, experiments, and theory, and is often invoked to explain phenotypic diversification within populations, some forms of speciation, and adaptive radiation. However, some foraging models predict the opposite outcome, and it therefore remains unclear whether competition will promote or inhibit niche expansion. We conducted experiments to test whether competition changes the fitness landscape to favor niche expansion, and if competition indeed drives niche expansion as expected. Using *Tribolium castaneum* flour beetles fed either wheat (their ancestral resource), corn (a novel resource) or mixtures of both resources, we show that fitness is maximized on a mixed diet. Next, we show that at higher population density, the optimal diet shifts toward greater use of corn, favoring niche expansion. In stark contrast, when beetles were given a choice of resources, we found that competition caused niche contraction onto the ancestral resource. This presents a puzzling mismatch between how competition alters the fitness landscape, versus competition's effects on resource use. We discuss several explanations for this mismatch, highlighting potential reasons why optimality models might be misleading.

## Introduction

Adaptive radiation, defined by rapid speciation and ecological diversification, plays an important role in the evolution of biological diversity (Schluter 2000). Cases of adaptive radiation are often attributed to the joint effects of new ecological opportunity and the diversifying effect of intraspecific competition (Van Valen 1965), which are expected to drive a population to expand its niche to include previously unused resources. This niche expansion occurs because intraspecific competition reduces the availability of preferred resources, favoring individuals who use previously ignored resources that provide some relief from competition. The resulting selection gradient can drive niche expansion via genetic evolution (Bolnick 2001; Agashe and Bolnick 2010), phenotypic plasticity (Svanbäck and Persson 2009), behavioral niche shifts (Werner and Hall 1974; Svanbäck and Bolnick 2007), or a combination of these processes (Agashe and Bolnick 2012). When niche expansion entails genetic diversification, assortative mating can amplify phenotypic variation leading to the emergence of distinct ecotypes and perhaps speciation (Levene 1953; Taper and Case 1985; Burger and Gimelfarb 2004; Dieckmann et al. 2004). Density-dependent niche expansion is thus posited to play a causal role in ecological speciation (Feder et al. 1995) and adaptive radiation (Schluter 2000).

The expectation that competition drives niche expansion arises from foraging theory. In its simplest version, individuals are expected to specialize on a small set of high-value resources when food is abundant, bypassing alternative resources whose opportunity cost (time spent capturing or digesting rather than searching for more valuable resources) exceeds their value. However, when preferred resources become scarce (due to resource competition), the opportunity cost of low-value resources is reduced and individuals begin to use resources that were previously overlooked, leading to niche expansion with increased competition (Emlen 1966; MacArthur and Pianka 1966; Pulliam 1974; Stephens and Krebs 1986). Similar outcomes are seen when using other modeling formats including ideal free distributions (Fretwell and Lucas 1969; Sih 1998), adaptive dynamics (Ackermann and Doebeli 2004), habitat selection (e.g., Brown 1998), and optimality models (for an example, see Data S1; for a review of factors affecting the outcome of competition between specialists and generalists, see Wilson and Yoshimura 1994). Density-dependent niche expansion also has broad empirical support (Werner and Hall 1974; Bolnick 2001; Sih and Christensen 2001; Svanbäck and Bolnick 2007). For example, as populations of *Glenuroides japonicus* (ant lions) grow, an increasing proportion of individuals settle in coarse sand, rather than in preferred fine sand (Morisita 1952). Such niche expansion can lead to diversification if population diet breadth increases largely through increased among-individual variation, rather than increased individual diet breadth (Bolnick et al. 2003; Svanbäck and Bolnick 2005; Bolnick et al. 2007).

In contrast, a few studies have demonstrated that competition can instead drive niche contraction (Sih and Christensen 2001). For instance, exposure to competing conspecifics caused *Columba livia* (rock pigeons) to be more, not less, selective while foraging (Inman et al. 1987). Although many basic models predict that competition should promote niche expansion, incorporating constraints such as limited foraging time, digestive capacity, and multifarious nutritional needs can alter the fitness landscape such that niche contraction rather than expansion is predicted to be more beneficial (Belovsky 1978, 1986). For example, an individual faced with both time and digestive constraints might maximize energy intake by eating a mixture of two resources, rather than specializing on a single most-profitable food as assumed in simpler optimal foraging theory. Changes in resource availability due to competition can shift this optimal mixture in either direction (more specialized on one food, or more equal use of both), depending on the precise model formulation (for an example, see Data S2). Thus, depending on what model one chooses to invoke, competition can be expected to drive niche expansion or contraction, in turn potentially facilitating or inhibiting diversification. Importantly, all these models are predicated on the assumption that individuals adopt optimal foraging strategies within the boundaries of their constraints.

The bigger question, then, is whether animal behavior is generally optimal. A large body of previous work – especially for choice of oviposition sites in female insects – has addressed the broader issue of the expected relationship between preference for and performance on alternative resources (e.g., Thompson 1988; Singer and Thomas 1992; Singer and Parmesan 1993). Although in most species females do choose resources that maximize offspring performance, there are many examples of nonoptimal choice or lack of preference even when the alternative resources provide very different fitness benefits (see Gripenberg et al. (2010) for a recent meta-analysis). Such a disconnect between preference and performance could arise due to genetic, ecological, or behavioral constraints; or due to inconsistent or weak selection for preference during the animal's ecological and evolutionary history (see, e.g., reviews by Mayhew (1997) and Scheirs et al. (2005).

Together, the diversity of predicted outcomes of intraspecific competition and the potential for nonoptimal behavioral resource choice calls for a more nuanced view of the role of competition in evolutionary diversification. In particular, there are still very few experimental tests of how competition drives niche expansion (or contraction). Of these experiments, none have simultaneously evaluated how competition changes the fitness landscape, and how it changes actual resource use. Furthermore, existing studies exclusively use a binary experimental design (high/low competition) that does not elucidate the function relating competition to niche width. As a result, despite decades of attention surprisingly little is known about when and why competition might drive niche expansion or contraction, and whether the outcome actually optimizes fitness.

Our prior experiment with the red flour beetle *Tribolium castaneum* suggested that short-term niche expansion onto a novel resource is negatively associated with density (Agashe and Bolnick 2010). This negative relationship is contrary to the adaptive radiation models of niche expansion, but possibly consistent with the linear programming models of foraging, which also consider other constraints on resource use. These alternative models make opposing predictions as to how the optimal diet will change with density. We therefore empirically measured how competition in populations of *T. castaneum* influences the shape of the fitness landscape (slope and peak location). In a separate experiment, we measured how competition alters population niche width within and across generations, for a wide range of densities to elucidate the functional relationship between competition and niche breadth, allowing us to use our empirical data to fully parameterize an optimality model of how niche width should evolve. This experiment is unique because, by measuring both the density-dependence of the fitness landscape and the population resource use, we are able to document both what the population should do if it were to behave optimally, and what it actually does.

## Methods

### Study system

We carried out a series of laboratory experiments measuring relationships between population density, resource use, and fitness of the flour beetle *Tribolium castaneum*. Beetle primary stock populations were produced by combining 20 wild-type strains of *T. castaneum* obtained from the Beeman laboratory (Biological Research Unit, Grain Marketing and Production Research Center, Kansas). These strains had been reared in the laboratory on 95% wheat flour and 5% brewer's yeast for several decades (>250 generations), and so wheat flour is their ancestral resource. In contrast, corn flour (no yeast added) is a novel and comparatively low-value resource that confers lower fitness (Agashe et al. 2011). Organically produced flours and yeast used to rear beetles were obtained from a single supplier throughout the experiment. Beetles were maintained at 33°C (±1°C) and 60% relative humidity in a laboratory incubator. When beetles are presented with a mixture of these resources, the beetles use a mixture of wheat and corn, allowing several metrics of niche shift. First, any nonzero amount of corn represents niche expansion relative to their ancestral all-wheat diet. Second, more equal consumption of these resources represents an increase in diet diversity.

### Experiment 1: density-dependent fitness of pure and mixed diets

To measure how beetle density affects fitness, we initiated experimental populations in three habitats: two homogenous habitats containing 50 g ancestral resource (wheat flour, denoted W) or novel corn flour (C), and a heterogeneous habitat containing a patch of 25 g wheat adjacent to 25 g corn (WC) (Fig. S1). In the WC habitat, beetles could move freely between resource patches. Within each habitat treatment, we varied the density of experimental populations from 20 to 200 adult beetles, in increments of 10, per 25 g of W resource, with one replicate per combination of habitat and density (19 densities per habitat type, 57 populations total). Because we manipulated adult density per g of *wheat* flour rather than total flour, the C patch in WC populations represented an additional nutritional and spatial resource. We sexed all individuals and founded all populations with a 1:1 sex ratio.

We calculated mean per capita fitness (*r*) in each flour treatment and at each density. Fitness was calculated as the number of adult offspring divided by the number of adults in the founding generation. We quantified per capita fitness 3 weeks after removing founding adults from W populations, and 5 weeks after removing founding adults from C populations to accommodate the slower development of beetles on corn (Agashe et al. 2011). Most first-generation offspring had matured to either the pupal or adult stage during this period, while any second-generation offspring would still be eggs and were not counted. Three C populations (densities *N* = 20, 40, and 140) survived to the end of the experiment; all other C populations were lost due to fungal infections.

To test whether selection favors generalists (WC) or specialists (W or C alone), we calculated the ratio of mean fitness of populations held on WC versus on W (*r*_*wc*_*/r*_*w*_) at each density, and *r*_*wc*_*/r*_*c*_ at the few densities where C populations persisted. We then use regression to test whether each ratio (the relative fitness advantage of a mixed-diet strategy) changes with population density.

### Experiment 2: estimating the optimal diet at low and high densities

Whereas Experiment 1 evaluated just three diet levels (W, WC, C) at a wide range of density, Experiment 2 measured the fitness landscape by measuring fitness effects of each of many diet combinations, focusing on just low and high density. We mixed corn and wheat in varying proportions (0, 20, 40, 60, 80, and 100% C). Crucially, by thoroughly mixing the flours (instead of adjacent patches), beetles consume the flours in the available ratio rather than exerting choice (confirmed by stable isotope analyses, see below). We placed eggs from the stock population in each mixture, allowed these to hatch and mature for 3 weeks, and then collected between 11 and 20 mature mated females per treatment. Each female was isolated in a centrifuge tube with the same flour ratio she was reared in, and allowed to lay eggs. We counted the number of eggs laid by each female every 3 days for 24 days, and also recorded the lifespan of the female. We used quadratic regression to determine the relationship between per capita fitness (number of eggs laid per female) and the proportion of corn in the flour mixture. This experiment was repeated at high density, with 200 females in 50 g of flour. To facilitate comparison with our test for additive fitness at low density, after 3 weeks, we isolated 4 mated females from each crowded population, placed females individually on 1 g of fresh flour (again at their natal % C) in a 35-mm plastic petri dish, and recorded the number of eggs found in each petri dish every second day for a total of 8 days. Quadratic regression of fitness against the proportion of corn again measured the fitness landscape. Analyses were repeated for lifespan.

The optimal proportion of corn in the diet was determined by solving for the maximum of the function estimated by quadratic regression, at both low and high population density. To test whether the optimum at low density was significantly different than at high density, we shuffled observations across densities while retaining % corn information, then recalculated the difference in estimated optima to generate a null value. The observed difference in optimal was compared with 10,000 null differences.

### Experiment 3: effect of density on resource use

We next tested whether beetle density drives niche expansion (more corn use) and diet diversification (more even use of wheat and corn). We examined density's effect on adult beetle resource use in the first generation of the density treatment, as well as resource use by their offspring. As in Experiment 1, we again setup one replicate population per density (*N* = 20 through 200 beetles per population, in increments of 10). Unlike Experiment 1, we varied density only for W and WC environments, as C populations are frequently inviable. Importantly, because beetles can move freely between wheat and corn patches in the WC treatment, but the flours are not mixed, beetles can exercise choice in their relative use of each resource. Each population was stocked with the appropriate number of newly eclosed adult beetles. After maintaining each population for 2 weeks (during which time adults laid eggs), we removed all adults from each population. We randomly subsampled 30 adults per population to assay their relative use of wheat and corn by quantifying the stable carbon isotope ratios of their bodies (for density *N* = 20, we pooled adults from experiments 1 and 3 so that we could draw a sample of 30 adults).

Beetles were oven-dried at 50°C for 72 h, individually packed in tin capsules, and shipped to the UC Davis Stable Isotope Facility. The facility analyzes ^13^C and ^15^N isotopes using a PDZ Europa ANCA-GSL elemental analyzer interfaced to a PDZ Europa 20–20 isotope ratio mass spectrometer (Sercon Ltd., Cheshire, UK). We used carbon and nitrogen stable isotope ratios to estimate individuals' dietary niche (% corn in diet and degree of cannibalism). Corn and wheat flour have different carbon stable isotope ratios (*δ*^13^C_wheat + yeast_ = −23.37; *δ*^13^C_corn_ = −11.84). Because these isotope ratios are preserved in beetle tissues, we can calculate the proportion of wheat and corn in any individual beetle's diet. Within each population, mean *δ*^13^C of sampled beetles indicates average corn use (Agashe and Bolnick 2010).

We confirmed the utility of *δ*^13^C in measuring diet in three additional experiments. First, we fed beetles various mixtures of wheat and corn (increments of 20% corn, as in Experiment 2), with one replicate container with 5 beetles per mixture. By thoroughly mixing the flour types, beetles consume wheat and corn in the ratio we provide them. We measured *δ*^13^C of whole beetles and confirmed that *δ*^13^C of beetles varies linearly with % corn in the diet (linear model: *δ*^13^C = −24.169 + 0.113 × % corn; *R*^2^ = 0.95, *F*_1,28_ = 598.9, *P* < 0.001). In a second validation experiment, we confirmed that our 2-week sampling period was sufficient to allow stable isotope turnover in beetle tissues and that the turnover rate itself was not affected by density. We reared eggs and larvae entirely on wheat, then transferred 1-week old adults to a well-mixed 1:1 wheat–corn mixture, with either 20 or 200 adults per population (six populations per density). Every 3 days (for a total of 18 days), we destructively sampled one replicate population from each density treatment, using 5 individuals per population for isotope analysis. We found that for both density treatments, beetle *δ*^13^C rapidly converged on corn isotope ratios in under 2 weeks, and then remained stable thereafter (Fig. S2, no significant effect of isotope signature between high (*N* = 200) and low (*N* = 20) density populations, ANCOVA *P* = 0.289). Thus, measuring *δ*^13^C at 2 weeks provides an accurate measure of recent diet that is unbiased by prior diet and density. As a third validation step, we tested whether high density substantially reduces individual beetle tissue growth, reducing carbon uptake and altering carbon isotope ratios. Using data from a previous experiment (Agashe and Bolnick 2012), we first confirmed that the total carbon quantified during carbon isotope analysis is strongly positively correlated with beetle weight (Fig. S3A). We then analyzed beetles from the present experiment to test whether population density was associated with reduced total carbon (as a proxy for tissue growth). Instead, we found a weak but significant positive relationship between the total carbon in beetle bodies and population density (Fig. S3B), indicating that individual tissue growth is not inhibited by density, and thus density cannot inhibit carbon uptake. This surprising positive relationship is as yet unexplained, but may reflect increased allocation of resources to energy reserves rather than reproduction.

Egg cannibalism is an important feature of *Tribolium* biology (Sokoloff 1977; Agashe and Bolnick 2010) and may be adaptive in stressful habitats (Via 1999). Hence, cannibalism may also increase in response to high population density. Nitrogen stable isotope ratios (measured as *δ*^15^N) typically increase with trophic position (e.g., rate of cannibalism), because protein assimilation leads to enrichment of ^15^N. We therefore used *δ*^15^N as a measure of trophic position to test whether cannibalism rates vary with density or resource type. To correct for the fact that flour type also affects *δ*^15^N ratio, we carried out a separate calibration experiment in which we reared beetles without access to eggs (in wheat + yeast, beetle *δ*^15^N = 4.61 and *δ*^13^C = −24.07; in corn, beetle *δ*^15^N = 7.08 and *δ*^13^C = −11.65; *n* = 5 beetles). We then calculated the expected beetle *δ*^15^N of beetles given their corn versus wheat consumption (expected *δ*^15^N = 9.41 + 0.199 × *δ*^13^C). Any additional enrichment in ^15^N above this expectation is due to cannibalism, quantified as *δ*^15^N = *δ*^15^N_observed_ − *δ*^15^N_expected_ (Fig. S4). As we do not know the change in *δ*^15^N as a function of the number of eggs consumed, cannibalism was measured on a relative rather than an absolute scale.

To test whether second-generation larval behavioral acceptance of the novel corn resource varied as a function of founding population density, we sampled 25 larvae from one replicate of each experimental population at the same time that we sampled adults for isotopes (Fig. S1). Each larva was tested in isolation, in a 35-mm plastic petri dish containing adjacent patches of W and C. We placed each larva in a hollow plastic tube on the boundary separating the two resources, recording its location (in W or C patch) after 24 h as a measure of its resource preference.

### Follow-up experiment A: quantifying oviposition and egg cannibalism

To test whether the C patch in WC populations offered a density-dependent refuge from cannibalism, we setup populations at two densities (*N* = 30 or 100; six and four replicates each), identical to those in Experiment 3. To each flour patch we added 5% neutral red dye, a nontoxic vital stain that colors eggs pink (Rich 1956). After 40 h for oviposition, we counted pink eggs in each patch and returned adults and eggs to fresh flour that did not contain dye. After another 24 hours allowing cannibalism, we counted the number of surviving pink eggs in each patch to quantify cannibalism rate of pink eggs in each resource. Fresh eggs laid by females during this period were white, and therefore, fecundity did not confound the measurement of cannibalism. We used a quasipoisson GLM to test whether oviposition depends on flour type, population density and their interaction.

### Follow-up experiment B: testing for differences in perceived value of resources

To determine whether beetles perceived density-dependent degradation of C differently than W, we first prepared conditioned flour by keeping adult beetles at high density (200 adults per 50 g flour) for 3 weeks. We sifted this “conditioned” flour to remove individuals of all stages. We founded five replicate high-density populations (*N* = 200) in containers with 25 g each of conditioned W and C, or 25 g each of fresh W and C (control populations). After 40 h, we removed and counted adult beetles from each flour type. We then thoroughly mixed flour from each patch and counted the number of eggs in a 1 g sample per patch.

## Results

### Experiment 1: density-dependent fitness of pure and mixed diets

**Figure 1:**
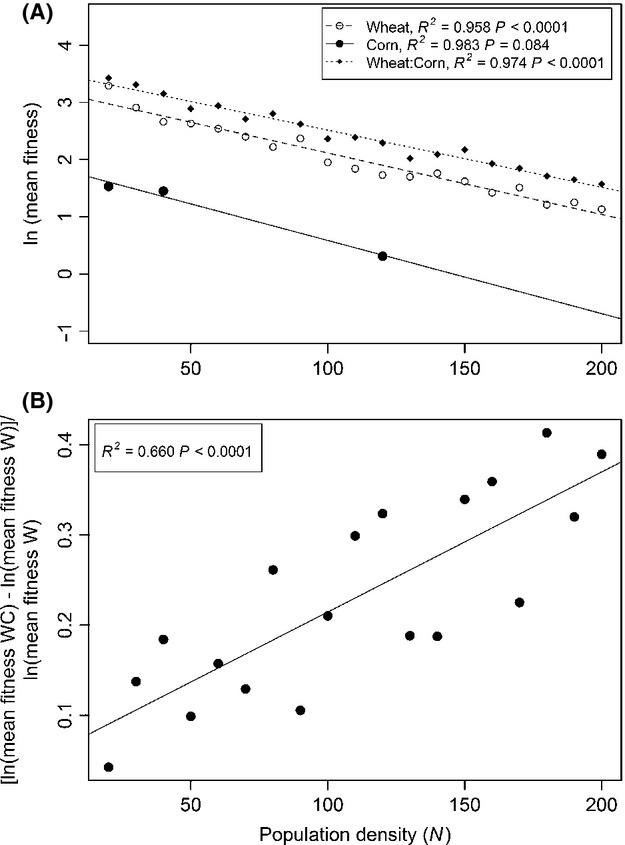
(A) Effect of beetle population density (*N*) on log fitness (number of adult offspring per adult beetle) at a range of adult densities, on wheat, corn, or a mixture. Using results in A as a measure of density-dependent fitness, we calculated the ratio of fitness in mixed wheat:corn habitat to fitness on wheat only. This fitness is plotted against population density (B) to measure the effect of competition on the relative advantage of a mixed diet.

As expected, competition reduces mean fitness in all resource types tested (per capita number of offspring). When examining log fitness, the decline of fitness with density is strikingly linear (Fig.1A), with effectively identical slopes in wheat, corn, and a combination of wheat and corn (in adjoining patches). The effect of competition on fitness is significant in W and WC, and marginally significant in C despite only three populations surviving to be censused (other populations had a fungal infection).

For all densities, beetles with a mixed diet (WC environment) had higher fitness than either W or C specialists (Fig.1A). This advantage of a mixed diet is consistent with prior research showing that these flours have complementary nutritional components (Sokoloff et al. 1966; Lecato 1973). The absolute value of this advantage is essentially constant for all densities, but the relative fitness advantage increases dramatically with density (Fig.1B, *P* < 0.0001). We conclude that niche expansion to include the novel corn resource is favored by selection at all densities, but this selection is stronger as density increases. This empirical result can be used to parameterize an optimality model (Data S1), which predicts that competition should drive increased use of corn (increased niche width) and greater diet evenness. Experiment 1 thus corroborates the view that competition should drive niche expansion.

### Experiment 2: estimating the optimal diet at low and high densities

**Figure 2:**
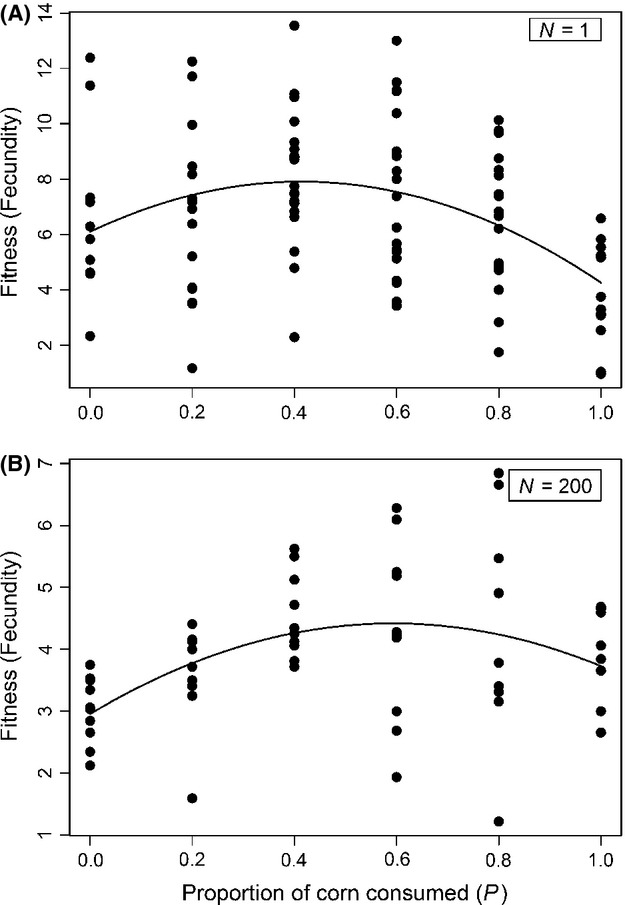
Effect of resource use on fitness. Female fecundity (eggs laid per day) as a function of increasing proportion of corn in supplied flour, at (A) low (*N* = 1 beetle per population, *n* = 10 to 19 females per flour mixture treatment) and (B) high density (*N* = 200 beetles per population, *n* = 10 females per flour mixture treatment).

Experiment 1 shows that a mixed diet confers higher fitness than a pure wheat or corn diet, but does not indicate what ratio of wheat or corn is optimal. To determine the optimal diet, we used quadratic regression to evaluate how fitness (average daily fecundity and lifespan) of single beetles (*N* = 1) varies with various ratios of thoroughly mixed wheat and corn. The result is an estimate of the fitness landscape, describing how fecundity varies as a function of resource use at each of two densities. We found a negative quadratic relationship between per capita fitness and % corn (Fig.2A; GLM, best model includes significant positive linear (*P* < 0.01) and negative quadratic (*P* < 0.001) terms). Individual beetles' fitness is maximized by a mixed diet with ∼41% corn. The relationship was similar when we estimated lifetime fecundity (Fig. S4), which accounts for both daily fecundity and longevity.

When we repeated this experiment at high density (*N* = 200 females per container), we again found a significant quadratic relationship between fecundity and % dietary corn (Fig.2B; GLM, best model includes significant positive linear (*P* < 0.01) and negative quadratic (*P* < 0.01) terms). Notably, at this higher density, the optimal diet entailed a significantly higher proportion of corn than at low density (59.29% compared to 41.35%; permutation test *P* = 0.0406). Although the optimal proportion of corn increased with density, the two densities (which bracket the values used in Experiments 1 and 3) confer essentially equal resource diversity being symmetrical around 50% wheat/corn. Thus, this experiment agrees on all counts with the results of Experiment 1: a mixed diet is favored by selection, and competition favors using an increased proportion of corn (niche expansion).

### Experiment 3: effect of density on resource use

Experiments 1 and 2 both predict that a mixed diet is always favored, but that competition should drive increased use of corn. To test these predictions, we conducted a third experiment in which we exposed individuals to a range of densities and WC resources and assayed the resulting shift in resource use of both first- and second-generation individuals. We found three distinct lines of evidence that competition drove decreased use of corn.

**Figure 3:**
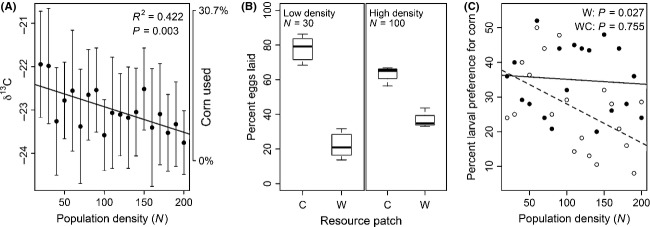
Evidence for density-dependent niche contraction. (A) Population mean adult carbon isotope ratio (±1SD, right-hand *y*-axis shows the corresponding proportion of corn in the diet) as a function of population density (total 19 populations). (B) Median oviposition in wheat versus corn (boxes show 25^th^ and 75^th^ percentiles; whiskers minimum and maximum values) at low (*N* = 30, *n* = 6 replicates) and high (*N* = 100, *n* = 4 replicates) density. The number above each box represents percent eggs laid in each habitat. (C) Proportion of offspring larvae preferring corn to wheat (dotted line and open circles = wheat populations; solid line and filled circles = wheat + corn populations; 19 populations per habitat treatment).

First, using stable carbon isotope signatures, we found that adult beetles used ∼20% corn at low density (Fig.3A). This mixed diet is consistent with the observation from Experiments 1 and 2 that a mixed diet is favored by selection even at low density. However, the observed proportion of corn in the adult beetles' diet decreased with density (*R*^2^ = 0.422, *P* < 0.003, Fig.3A). This directly contradicts the expectation that competition should drive increased corn use, arising from Experiments 1 and 2. Importantly, Experiment 3 demonstrates a within-generation change in foraging, presumably representing a shift in foraging behavior rather than evolutionary change. That said, in models of ideal free distributions and optimal foraging, the presumption is that individuals' foraging behaviors are changed to maximize fitness. Thus, changes in foraging behavior should follow the peak of the fitness landscape and thus resemble and anticipate longer-term evolutionary changes.

Second, we were surprised to find that females always preferred to oviposit in corn, despite wheat being the ancestral resource. However, females' oviposition rate on corn decreased with density (quasipoisson GLM, best model: density: *P* < 0.001; parents' resource: *P* < 0.001; density × resource: *P* < 0.01; Fig.3B).

Third, resource choice assays revealed that larvae from wheat populations increasingly preferred wheat at high density (*R*^2^ = 0.257, *P* = 0.03, Fig.3C). It is important to note that these wheat-reared larvae were completely naïve to corn, as neither they nor their parents had encountered the novel resource. Thus, stronger intraspecific competition induced individual niche contraction in individuals that were naïve to the novel resource and did not directly experience the competitive dual-resource environment. Interestingly, this density-dependent effect was not observed for larvae from WC populations (*R*^2^ = 0.006, *P* = 0.76, Fig.3C; generalized linear model (GLM) with binomial errors: preference ∼ density + flour × density; for density, *P* < 0.001, for flour × density, *P* = 0.002). The lack of effect for WC larvae is surprising, given that their parents exhibited density-dependent niche contraction (Fig.3A). Nonetheless, these three lines of evidence show that both larval and adult beetles increasingly use wheat as density increases, both for feeding (adults and larvae) and for oviposition by adult females.

There is some among-individual diet variation at all densities, as exhibited by variation in the proportion of corn versus wheat use within experimental populations. The amount of among-individual variation is not significantly affected by competition (Fig. S6). However, the trend is for diet variation to decline with competition, a result also observed in some natural populations (Jones and Post 2013), but contrary to other populations where competition drives increased diet variation among individuals (Swanson et al. 2003; Svanbäck and Bolnick 2007; Araujo et al. 2008; Martin and Pfennig 2009).

### Testing possible causes of density-dependent niche contraction

The previous results indicate that selection favors greater corn use with increased density (Experiments 1 and 2), but beetles actually did the opposite when given a choice. Here, we report the results of follow-up experiments designed to test various hypotheses that might account for this contrarian result.

First, we tested whether beetles might have indeed expanded their resource use with greater competition, but did so via increased cannibalism rather than increased corn use. Cannibalism of eggs and pupae is common in *Tribolium* (Sokoloff 1977; Via 1999), providing a third resource that may be increasingly exploited with increasing population density. However, we find no evidence for density-dependent niche expansion across trophic levels (Experiment A). The adult stable nitrogen isotope ratio, which indicates trophic position (Hobson and Clark 1992), was not associated with population density (*R*^2^ = 0.0467, *P* = 0.374, Fig. S5A). Experimentally measured egg cannibalism rates were also uncorrelated with population density (binomial GLM, best model includes only a resource effect, *P* < 0.001; Fig. S5B). Because cannibalism rates are density-independent in our experimental system, trophic-level niche shifts cannot explain the discrepancy between model and experimental results.

Second, it is possible that mixed diet indeed confers higher fitness at high density, but that at high density beetles perceive corn as being particularly noxious. In other words, beetles might exhibit density-dependent aversion to corn for reasons unrelated to its nutritive value. For instance, beetles secrete waste products and toxic quinones that accumulate in flour. Although fitness declines at approximately the same rate with density on each resource (Fig.1A; *t*-test on regression slopes: *t* = 1.612, *P* = 0.124), noxious secretions might be more abundant or readily detected in corn than in wheat. If so, at a given population density, beetles may inaccurately perceive corn as being disproportionately more detrimental than wheat, causing niche contraction at higher density. To test this idea, we gave beetles a choice between fresh resources, or between “conditioned” resources (both previously exposed to an equally high density of beetles; Experiment B). The relative abundance of adults and oviposition in each resource did not vary with flour quality (Fig. S6; adult abundance, *t*-test: *t* = 0.507, *P* = 0.634; oviposition, *t*-test: *t* = 0.0826, *P* = 0.937). Therefore, we conclude that *Tribolium* are not disproportionately averse to corn at a given level of crowding.

Third, it is possible that niche expansion occurs only for some individuals, whereas the majority of individuals retrench on a familiar resource (Bolnick et al. 2007). In this case, we expect competition to increase the among-individual diet variation even as mean corn use declines. However, we found no relationship between the coefficient of variation in corn use and population density (Fig. S7).

Fourth, we considered the possibility that trade-offs between using the two resources reduce the fitness of generalists. Trade-offs in resource use are found frequently in insects (e.g., Lee et al. (2009) and may arise in two ways. First, fitness on W and C could be negatively correlated across genotypes (W-adapted genotypes are less fit on C and vice versa). However, data comparing multiple *Tribolium* strains do not support a negative genetic correlation between fitness on alternate resources (Agashe et al. 2011). Second, using one resource might undermine individuals' ability to compete for the alternate resource (Persson 1985; Lewis 1986; Ackermann and Doebeli 2004). In this case, generalists would be poor competitors against specialists, generating a density-dependent penalty for eating corn. However, the observation that a mixed diet is optimal (Experiments 1 and 2), even at high density, argues against such fitness penalties against generalists.

## Discussion

Numerous experiments and observational studies support the notion that intraspecific competition tends to drive population niche expansion (Van Valen 1965; Bolnick 2001; Svanbäck and Bolnick 2007; Martin and Wainwright 2013). A smaller number of studies have directly assayed how competition favors niche expansion by altering the shape of the fitness landscape (Schluter 2003; Bolnick 2004; Martin and Pfennig 2009). These results support a long-standing body of theory suggesting that negative frequency-dependent competition within a population will tend to favor individuals that can access atypical resources that are currently under-used, thereby driving either directional or disruptive natural selection (Rosenzweig 1978; Doebeli 1996; Ackermann and Doebeli 2004; Svanbäck and Bolnick 2005). Our experimental measures of fitness landscapes fit well within this broader literature, in that we find that greater competition drives stronger selection for a mixed diet that includes a novel resource. This is supported by two separate experiments (1 and 2), one varying density continuously for three different resource use patterns, the other varying resource use continuously for low and high density.

In stark contrast, when we varied density and allowed beetles to choose their resource use, we found exactly the opposite result: a clear case of behavioral density-dependent niche contraction in *Tribolium castaneum* beetles exposed to a novel resource. This niche contraction is contrary to classical expectations that underlie theories of adaptive radiation, and contrary to our own empirically parameterized optimality model (Data S2). There are some forms of foraging models that can generate density-dependent niche contraction (illustrated in Data S2), in agreement with the result of Experiment 3. Namely, linear programming models of multiple simultaneous trade-offs (such as digestive and time constraints) can, depending on details of the trade-offs, predict density-dependent niche contraction. However, these linear programming models are still optimal foraging models, which suggest that individuals adopt strategies that maximize their fitness. Niche contraction is therefore expected because competition causes the optimal diet to shift toward specialization on one resource. In contrast, in our study the observed niche shifts (Experiment 3) are in the opposite direction of what seems to be optimal (Experiments 1 and 2). We are therefore left with a puzzling inconsistency between what the beetles should do (given selection), and what they actually do.

We evaluated numerous hypotheses that might have explained this inconsistency between what we expect given the fitness landscape, and what beetles actually did (Table1). None of these explanations were supported by follow-up experiments. First, the advantages of niche expansion onto corn at high density could be negated by disproportionately intense egg cannibalism on corn at high density. Thus, beetles might avoid foraging (and ovipositing) on corn to improve survival of their eggs, thus driving niche contraction onto wheat. This hypothesis is doubly invalidated, because we found (1) no increase in cannibalism with density; (2) higher cannibalism on wheat; and (3) oviposition preference for corn. Second, although competition degrades the fitness value of wheat and corn at equal rates (Fig.1), perhaps beetles on corn are more sensitive to olfactory cues associated with competition, and thus disproportionately avoid corn as density increases. We found no support for this hypothesis: beetles kept at low density but given a choice between previously degraded wheat and corn did not disproportionately avoid corn. Finally, we found no evidence for trade-offs, which could cause individuals consuming both resources to become poor competitors on any one resource. Such trade-offs could arise, for instance, if there are appreciable travel costs that reduce the fitness of generalists who regularly switch between habitat patches. We consider this unlikely, because beetles moved extensively across both resources at all densities in our experiments. If travel were costly we would instead have expected dispersal between patches to decline with density. Also, data from a previous experiment with the same spatial setup (Agashe 2009) showed no association between population density and adult distribution across patches (Fig. S8). Finally, generalists were more, not less, fit (Experiments 1 & 2), inconsistent with strong trade-offs.

**Table 1:** Hypotheses tested in this study. After our main hypothesis of density-dependent niche expansion was rejected (first row), a set of hypotheses (following rows) were proposed and tested to explain our finding of density-dependent niche contraction.

Hypothesis tested	Experimental test	Outcome
Density-dependent niche expansion from ancestral (wheat) to alternate resource (corn)	Experiment 3 – Test of the effect of density on: (i) adult dietary resource preference (ii) female oviposition resource preference (iii) larval dietary resource preference	Results are consistent with density-dependent niche contraction: (i) The proportion of corn in adult beetle's diet decreased with density (Fig.3A) (ii) Female oviposition on corn decreased with density (Fig.3B) (iii) Larvae raised on wheat increasingly preferred wheat at high density (Fig.3C)
Density-dependent niche expansion on alternate resource (via cannibalism)	Experiment A – Test of the effect of density on rate of cannibalism	Rate of cannibalism is density-independent (Fig. S5A and B)
Density-dependent refuge from cannibalism	Experiment A – Test of the effect of density on rate of cannibalism in alternate resources	Rate of cannibalism on both resources is density-independent (Fig. S5A and B)
Difference in density-dependent degradation of alternate resources	Experiment B – Test of the effect of density on perceived resource quality for: (i) adult resource preference (ii) female resource preference for oviposition	Adult preference for the alternate resources did not vary with their quality (Fig. S6A and B, respectively). Hence, the quality of both resources is perceived to degrade equally at high density.
Among-individual variation in resource use	Test of the effect of density on among-individual diet variation	No relationship between the coefficient of variation in corn use and density (Fig. S7)
Genetic trade-offs between the use of resources (two distinct specialist genotypes)	Comparison of fitness on alternate resources across different *Tribolium* strains (Agashe et al. 2011)	No evidence for negative genetic correlation between fitness on alternate resources (Agashe et al. 2011)
Performance trade-offs between the use of resources (cost of generalization)	Experiments 1 and 2 – Comparison of fitness of beetles on a range of diet mixture	Mixed diet is found to be optimal, even at high density, arguing against a fitness penalty for generalists (Fig.2A and B)

**Figure 4:**
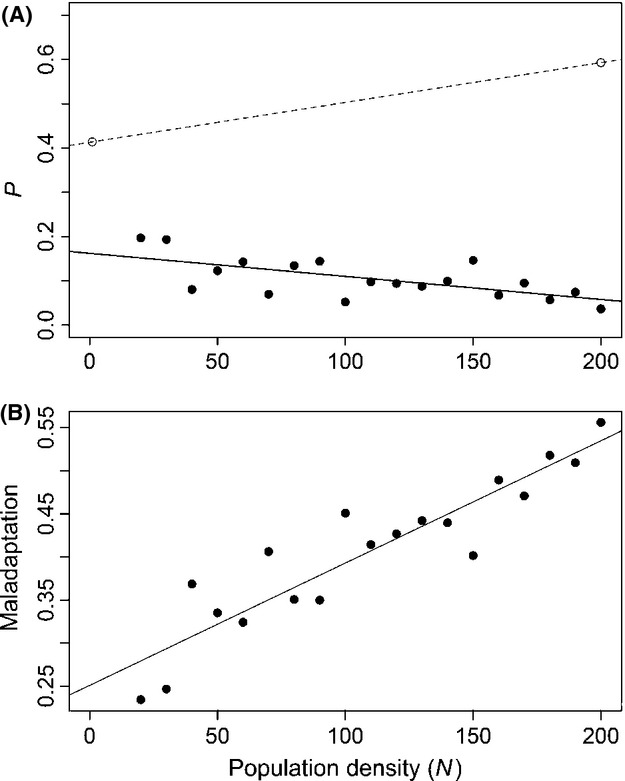
Expected optimal and observed resource use. (A) Optimal corn use from fitness assays (dotted line, open circles, *n* = 2) and actual corn use in populations (solid line and circles, *n* = 19) as a function of population density. (B) Deviation from optimality (measured as the difference between optimal and actual corn use) increases with population density (*n* = 19).

We propose another possible resolution for the apparent contradiction between how competition alters selection for resource use, versus how resource use actually changes. If we accept that Experiments 1 and 2 yield accurate reflections of how competition affects the fitness landscape, we must conclude there really is a fitness advantage of consuming a mixed diet and using more corn at higher density. Thus, instead of looking for explanations why the optimal percent corn should decline with competition, we could accept that the optimum does indeed increase with competition. From this point of view, it appears that our beetles acted nonoptimally (decreasing % corn use), and the resulting deviation between the optimal and actual diet becomes larger with increasing competition (Fig.4). Such density-dependent maladaptation could occur if stress from competition reduces individuals' cognitive abilities (Graham et al. 2009) needed to choose the best proportion of resources. Alternatively, low energy levels can reduce individuals' capacity to sample alternate habitats and gather information about the relative value of each patch. At present, this is just a speculative post hoc explanation. However, our data clearly show that competition leads to an increasing deviation between the optimal and actual diets (Fig.4B). Such density-dependent deviation from optimal foraging is a potentially general phenomenon, if competition reduces rational decision-making abilities. As a result, competition could reduce population mean fitness not just via direct constraints on resource intake, but indirectly by inducing maladaptive behavior. We thus find a peculiar case of nonoptimal resource choice in beetles, where adult behavioral choices (for feeding or oviposition) do not track density-dependent change in the optimal ratio of the two resources.

Although the literature on adaptive radiations is replete with discussion of the diversifying effect of competition, our study is not alone in finding that competition reduces niche breadth. A recent study on natural and experimental populations of alewife fish also shows evidence for population niche contraction and individual specialization under increased competition (Jones and Post 2013). When experimental populations at high density were supplemented with additional zooplankton; however, the niche contraction effect disappeared. The authors suggest that niche contraction was due to strong effects of alewife on their resource base (“strong interaction strength”), and when this effect is artificially decreased, niche contraction does not occur. Thus, effective competition is a function of both the consumer density and the degree to which consumers deplete resources (independently of density). In practice, separating these two is difficult, especially in our system where resource supplementation without altering beetle density is impossible. However, the alewife study points to a general mechanism (strong effects of consumers on resource base) by which increasing competition may prevent niche expansion in natural populations.

In conclusion, we found clear experimental evidence that competition can drive selection for greater use of a novel resource. However, we also found evidence that competition instead drove reduced use of the novel resource. At present, we have no specific explanation for this apparently maladaptive behavior, although we are able to reject several proposed mechanisms and can propose several potential explanations. Regardless of the precise mechanism, it is clear that competition can sometimes drive niche contraction. Such niche contraction is significant because it undercuts the generality of ecological explanations for adaptive radiation and speciation. The implication is that the ecological theory of adaptive diversification may be more limited than previously believed. It is thus an open question whether intraspecific competition typically promotes or constrains diet diversification within populations, and how strongly it affects ecological speciation.

## Supporting Information

**Figure S1.** (A) Experimental design with one density and habitat treatment detailed in each case for clarity.

**Figure S2.** Low (black) and high (blue) competition populations (*N* = 20 and 200, respectively) exhibit similar rates of isotopic turnover, following exposure to a novel resource.

**Figure S3.** Testing for reduced total beetle food intake as a function of density.

**Figure S4.** Density independent female fitness, measured as lifetime fecundity (the product of female fecundity (eggs laid per day) and lifespan (in days)), as a function of increasing proportion of corn in supplied flour.

**Figure S5.** (A) Population mean adult nitrogen isotope ratio as a function of adult population density.

**Figure S6.** (A) Mean proportion of adults and (B) number of eggs laid in C (in a two-patch habitat with novel C and ancestral W resource), when resources are conditioned versus when they are fresh (5 replicates per treatment).

**Figure S7.** No effect of population density on population (A) variance and (B) coefficient of variation in the proportion of corn included in the diet (*D*).

**Figure S8.** No effect of population density on adult beetle presence in wheat and corn patches (using biweekly census data collected for 42 populations across 9 months, from experiments described in Agashe (2009)).

**Data S1.** Optimality model of density-dependent resource use.

**Data S2.** Linear programming models of optimal foraging theory.
