# Supplementary material for: Intraspecific competition reduces niche width in experimental populations
Source: Ecol Evol. 2014 Sep 30;4(20):3978–90. doi: 10.1002/ece3.1254 (PMC4242580; doi:10.1002/ece3.1254)
Supplement: Supplementary file 1 — Figure S1. (A) Experimental design with one density and habitat treatment detailed in each case for clarity. [file ece30004-3978-SD1.docx]

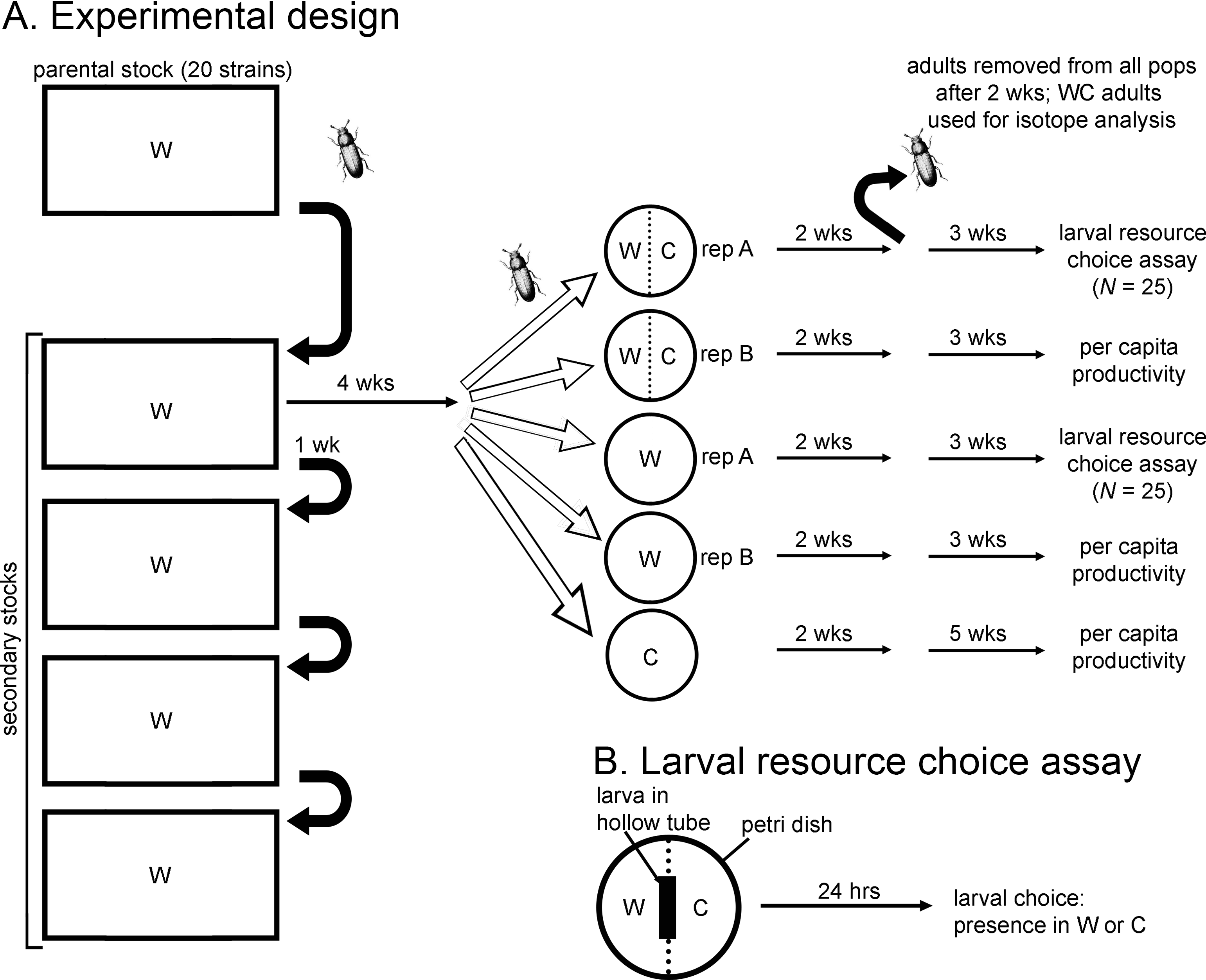


**Figure S1.** (A) Experimental design with one density and habitat treatment detailed in each case for clarity. Filled block arrows indicate transfer of the entire adult population, open block arrows indicate transfer of a fraction of the adult population, and single line arrows indicate waiting times. (B) Larval resource choice assay (see Methods in main text). Since it was not feasible to handle all experimental populations at the same time, we used the primary stock population to sequentially initiate four secondary stocks at one-week intervals: (1) adults from the primary stock were randomly chosen to initiate a first secondary stock, (2) after one week of egg laying, the same adults were transferred to a new secondary stock to lay eggs for one week, and so on to create four secondary stocks of beetles that were one week apart in age but belonged to the same generation started from the same parents. All experimental populations were founded with adult beetles emerging from the secondary stock populations four weeks after being initiated. To prevent any systematic error due to maternal age-related effects, we initiated disparate density treatments from each batch of parents. E.g. adults from the first batch of secondary stocks were used to initiate experimental populations at density 20, 50, 80, 110, 140 and 200; the second batch was used for densities 30, 60, 90, 120, and 150 and so on. We initiated two replicate populations at each density (for W and WC habitats), using one replicate to estimate productivity and the other to measure dietary niche.
