# Supplementary material for: Intraspecific competition reduces niche width in experimental populations
Source: Ecol Evol. 2014 Sep 30;4(20):3978–90. doi: 10.1002/ece3.1254 (PMC4242580; doi:10.1002/ece3.1254)
Supplement: Supplementary file 2 — Figure S2. Low (black) and high (blue) competition populations (N = 20 and 200, respectively) exhibit similar rates of isotopic turnover, following exposure to a novel resource. [file ece30004-3978-SD2.docx]

**Figure S2.** Low (black) and high (blue) competition populations (*N* = 20 and 200, respectively) exhibit similar rates of isotopic turnover, following exposure to a novel resource. Six replicate populations of beetles at each density treatment were initiated on wheat, and then transferred as adults to WC. Isotope signatures were measured in 5 beetles per treatment at day 0 (before the transfer), and every subsequent 3^rd^ day until day 18. Isotope signatures converge on a relatively stable value between five to 12 days after initial transfer. In Experiment 3 (main text) beetle isotopes were assayed at 14 days. The key point here is that differences in density do not influence the rate of isotopic incorporation, and isotope signatures at our final sample point in Experiment 3 are not appreciably affected by natal isotope ratios.
