# Supplementary material for: Intraspecific competition reduces niche width in experimental populations
Source: Ecol Evol. 2014 Sep 30;4(20):3978–90. doi: 10.1002/ece3.1254 (PMC4242580; doi:10.1002/ece3.1254)
Supplement: Supplementary file 3 — Figure S3. Testing for reduced total beetle food intake as a function of density. [file ece30004-3978-SD3.docx]

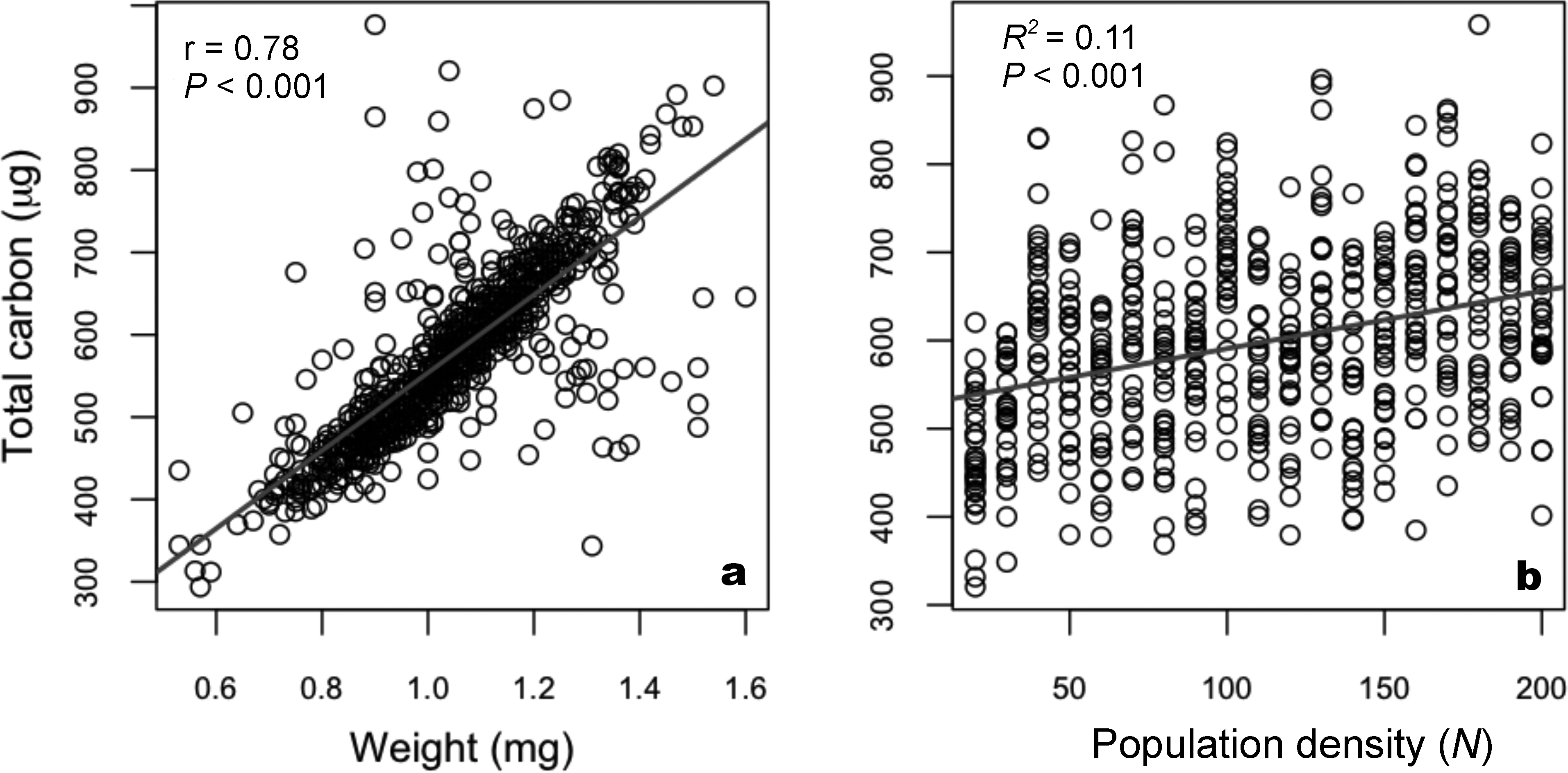


**Figure S3.** Testing for reduced total beetle food intake as a function of density. (A) Total carbon from mass spectrometry analysis of whole beetle samples for carbon isotope ratio, as a function of measured individual beetle weight. Data are from the experiments described in (Agashe and Bolnick 2012). (B) Total carbon in individual beetle samples from the current study, as a function of population density. Each data point is an individual beetle, and grey lines show best-fit regression lines.
