# Supplementary material for: Intraspecific competition reduces niche width in experimental populations
Source: Ecol Evol. 2014 Sep 30;4(20):3978–90. doi: 10.1002/ece3.1254 (PMC4242580; doi:10.1002/ece3.1254)
Supplement: Supplementary file 4 — Figure S4. Density independent female fitness, measured as lifetime fecundity (the product of female fecundity (eggs laid per day) and lifespan (in days)), as a function of increasing proportion of corn in supplied flour. [file ece30004-3978-SD4.docx]

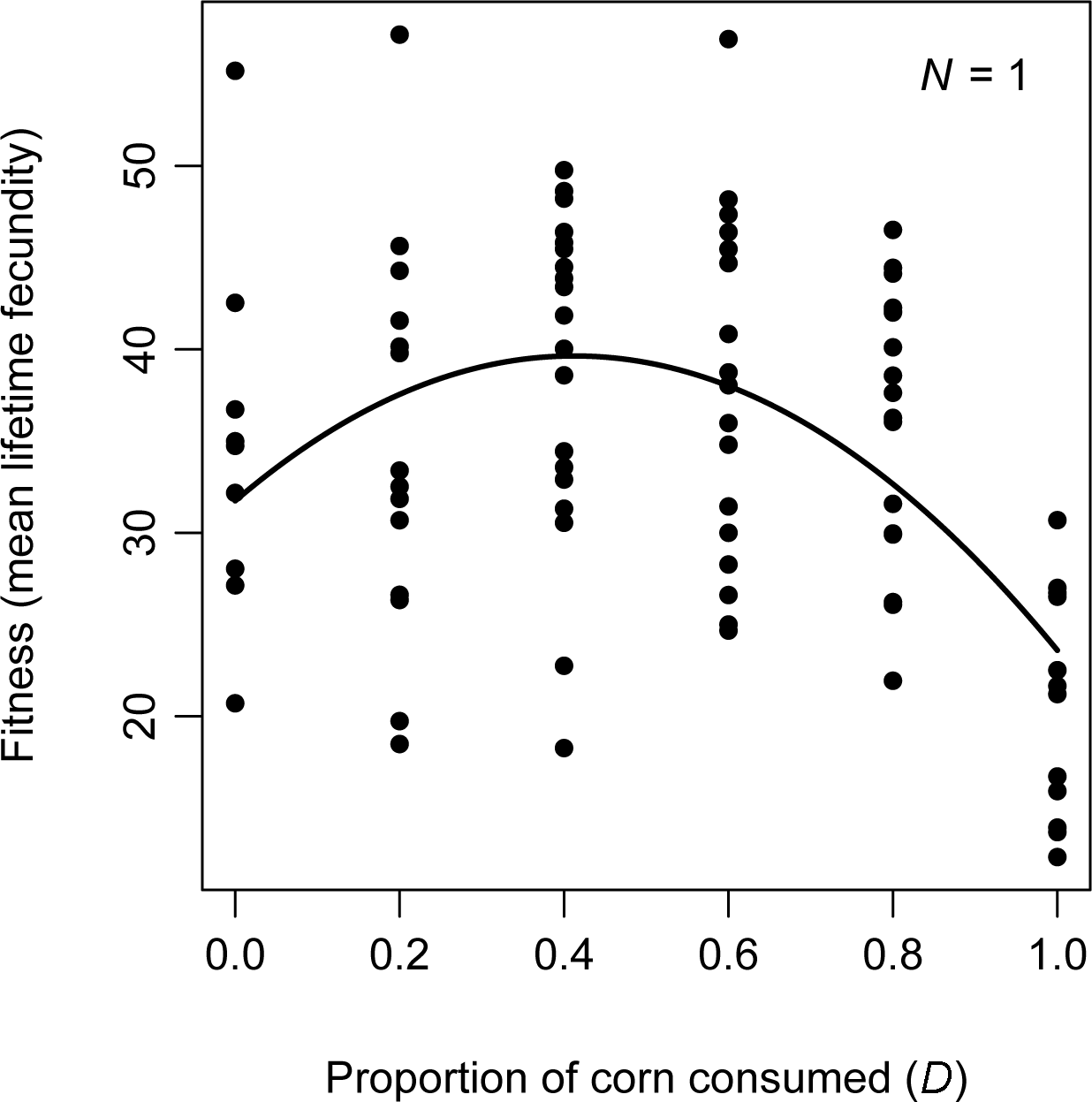


**Figure S4.** Density independent female fitness, measured as lifetime fecundity (the product of female fecundity (eggs laid per day) and lifespan (in days)), as a function of increasing proportion of corn in supplied flour.
