# Supplementary material for: Intraspecific competition reduces niche width in experimental populations
Source: Ecol Evol. 2014 Sep 30;4(20):3978–90. doi: 10.1002/ece3.1254 (PMC4242580; doi:10.1002/ece3.1254)
Supplement: Supplementary file 5 — Figure S5. (A) Population mean adult nitrogen isotope ratio as a function of adult population density. [file ece30004-3978-SD5.docx]

**
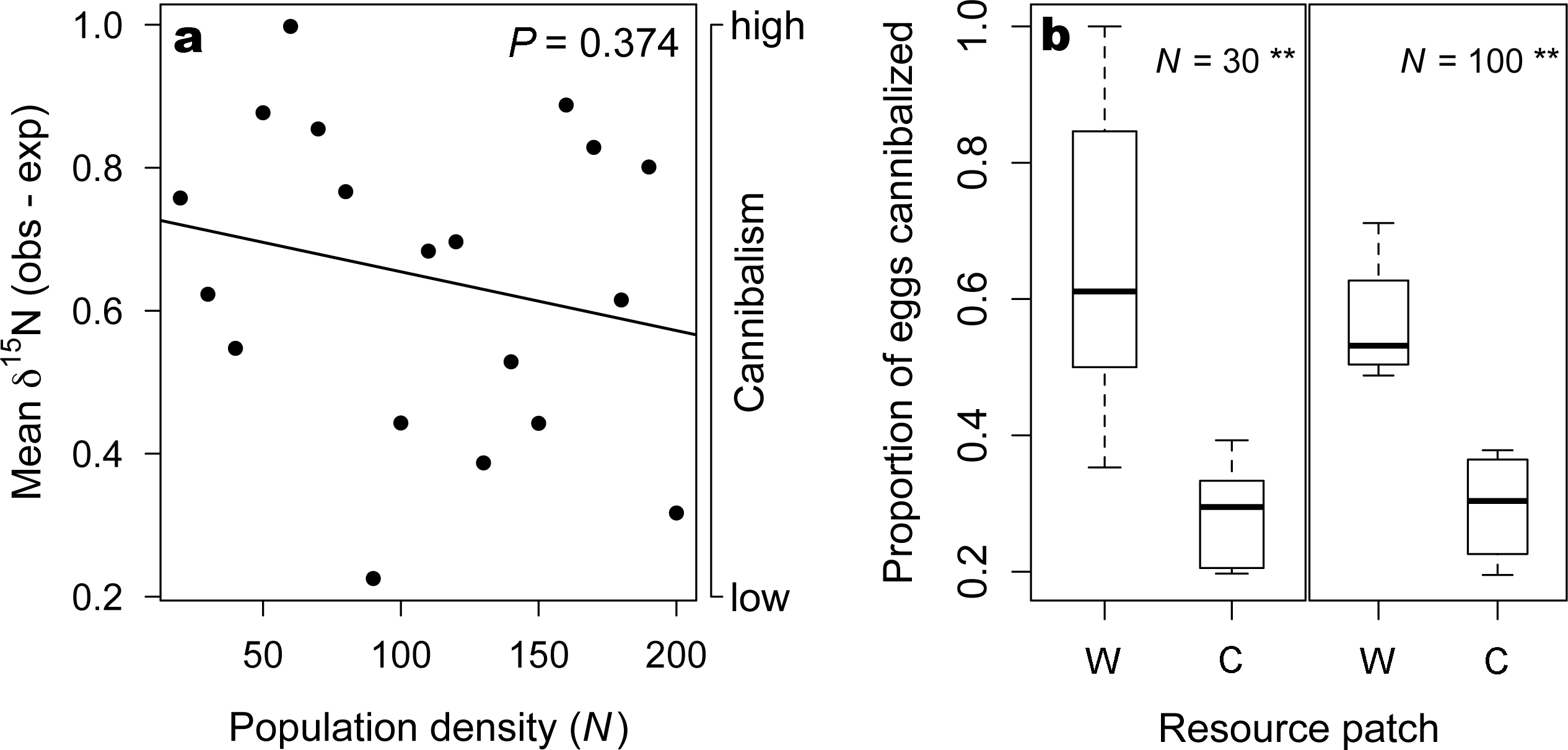
**

**Figure S5.** (A) Population mean adult nitrogen isotope ratio as a function of adult population density. Right-hand y-axis shows how the stable isotope ratio relates to the degree of cannibalism. (B) Box plots showing the median proportion of eggs cannibalized by adults in the ancestral wheat resource and novel corn resource patches in 24 hours (with boxes showing 25^th^ and 75^th^ percentiles, and whiskers the minimum and maximum data values), at low (*N* = 30, 6 populations) vs. high (*N* = 100, 4 populations) density. Asterisks denote significant (*P* < 0.01) patch effect.
