# Supplementary material for: Intraspecific competition reduces niche width in experimental populations
Source: Ecol Evol. 2014 Sep 30;4(20):3978–90. doi: 10.1002/ece3.1254 (PMC4242580; doi:10.1002/ece3.1254)
Supplement: Supplementary file 6 — Figure S6. (A) Mean proportion of adults and (B) number of eggs laid in C (in a two-patch habitat with novel C and ancestral W resource), when resources are conditioned versus when they are fresh (5 replicates per treatment). [file ece30004-3978-SD6.docx]

**
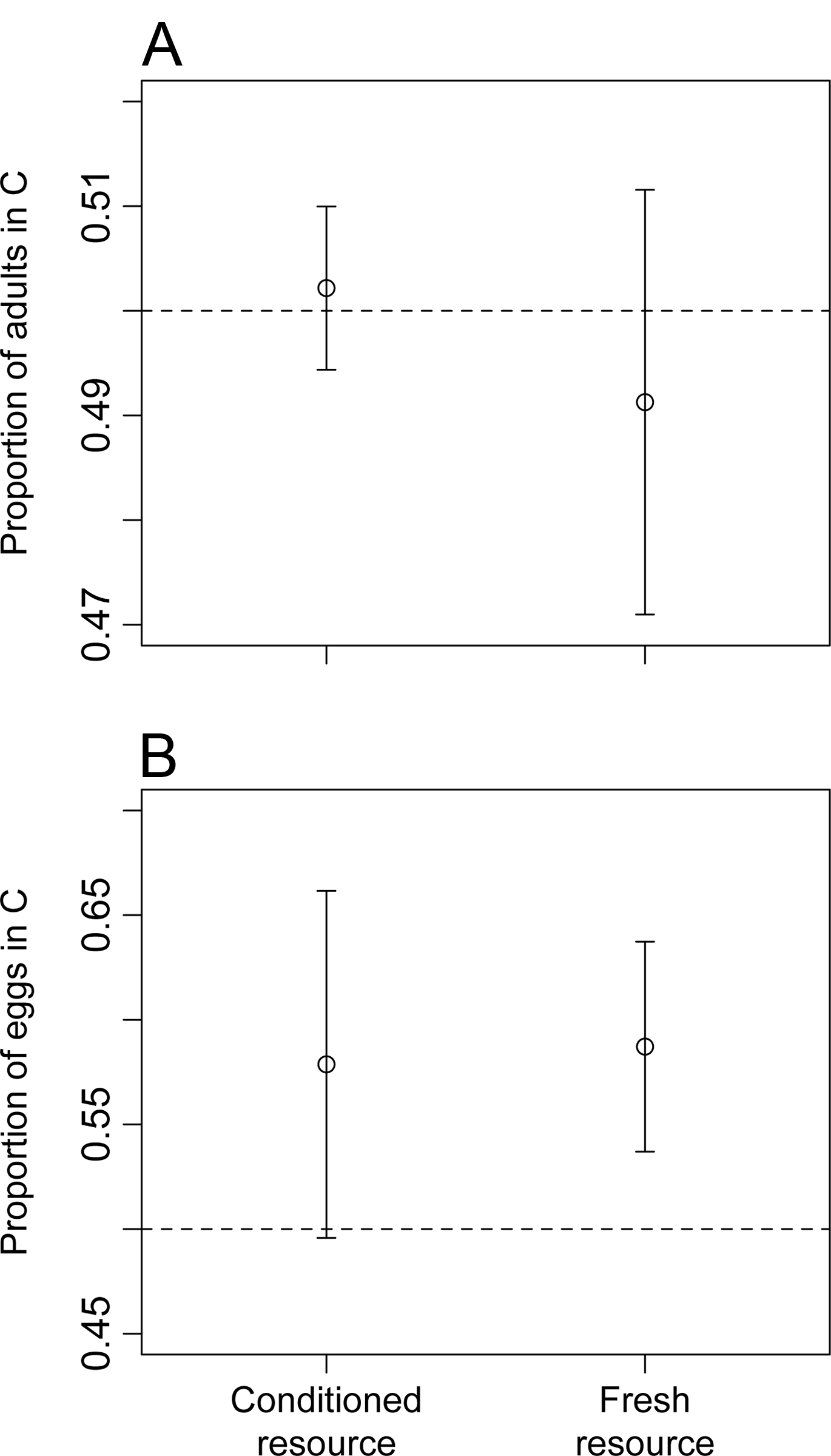
**

**Figure S6.** (A) Mean proportion of adults and (B) number of eggs laid in C (in a two-patch habitat with novel C and ancestral W resource), when resources are conditioned versus when they are fresh (5 replicates per treatment). The bars indicate ± 1 Standard Error, and the horizontal line indicates equal distribution of adults and eggs in each resource patch.
