# Supplementary material for: Intraspecific competition reduces niche width in experimental populations
Source: Ecol Evol. 2014 Sep 30;4(20):3978–90. doi: 10.1002/ece3.1254 (PMC4242580; doi:10.1002/ece3.1254)
Supplement: Supplementary file 7 — Figure S7. No effect of population density on population (A) variance and (B) coefficient of variation in the proportion of corn included in the diet (D). [file ece30004-3978-SD7.docx]

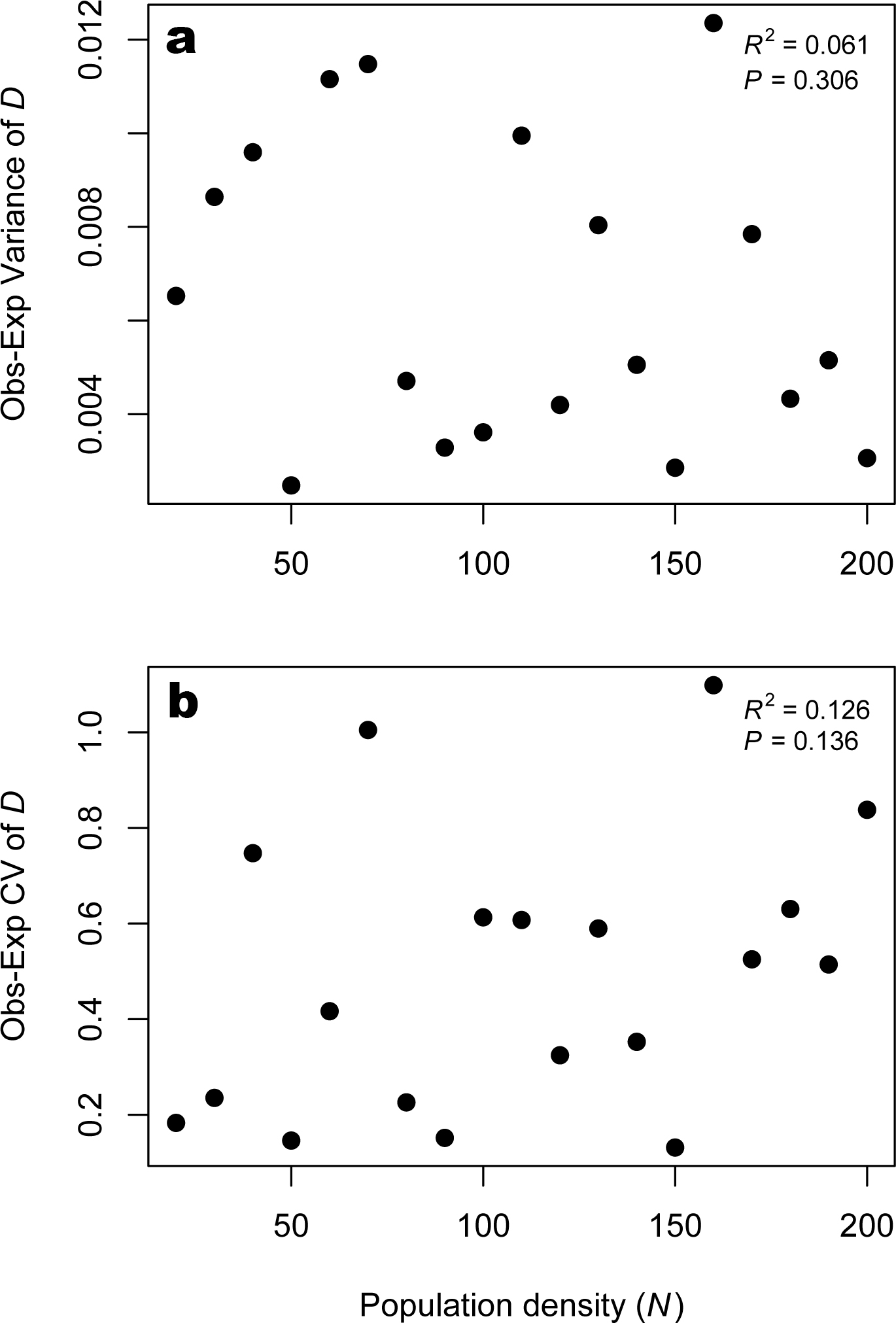


**Figure S7.** No effect of population density on population (A) variance and (B) coefficient of variation in the proportion of corn included in the diet (*D*). The y-axis is the difference between the observed and expected variance or covariance, given a binomial diet choice.
