# Supplementary material for: Intraspecific competition reduces niche width in experimental populations
Source: Ecol Evol. 2014 Sep 30;4(20):3978–90. doi: 10.1002/ece3.1254 (PMC4242580; doi:10.1002/ece3.1254)
Supplement: Supplementary file 8 — Figure S8. No effect of population density on adult beetle presence in wheat and corn patches (using biweekly census data collected for 42 populations across 9 months, from experiments described in Agashe (2009)). [file ece30004-3978-SD8.docx]

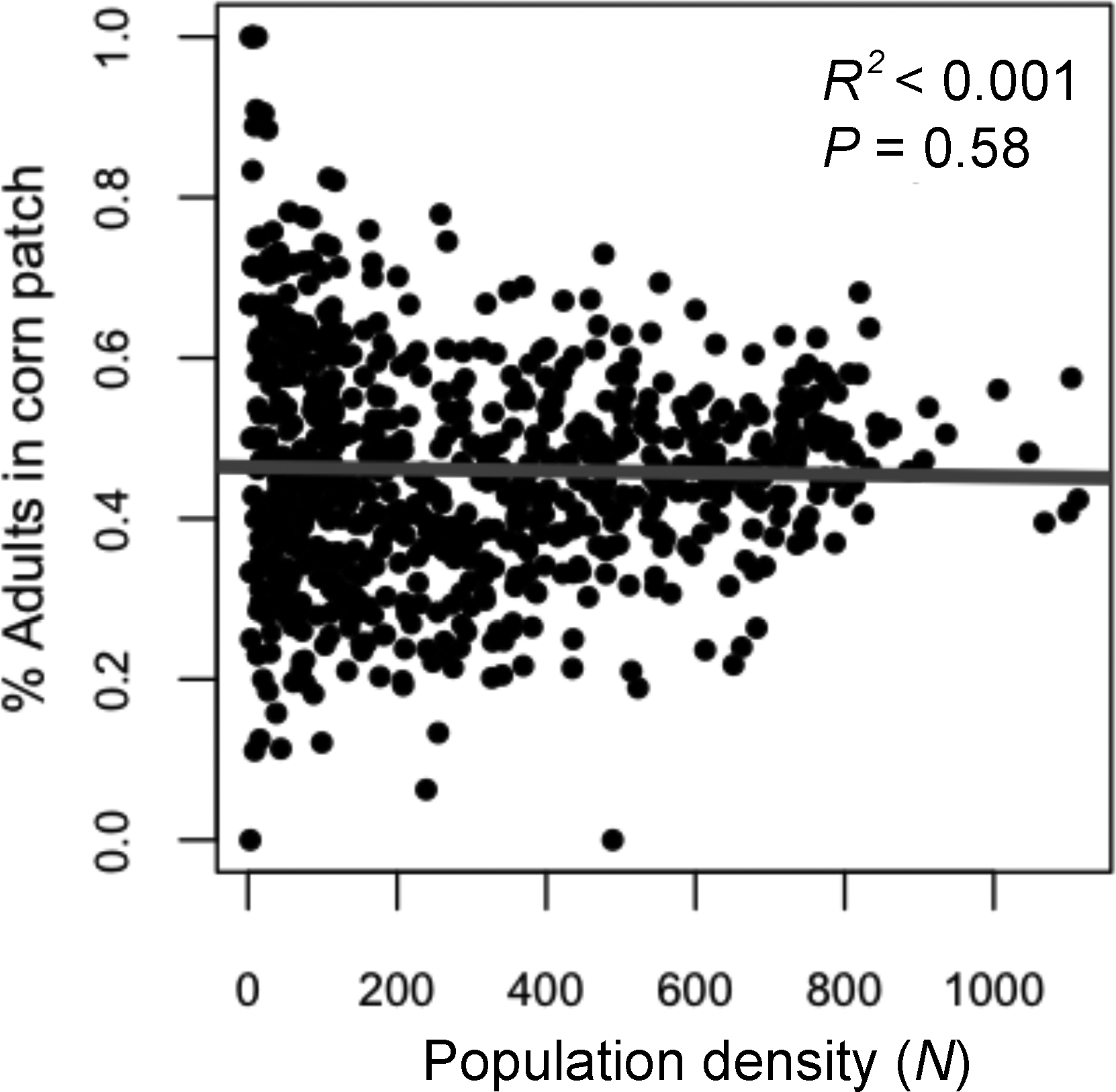


**Figure S8.** No effect of population density on adult beetle presence in wheat and corn patches (using biweekly census data collected for 42 populations across 9 months, from experiments described in Agashe (2009)).
