# Supplementary material for: Intraspecific competition reduces niche width in experimental populations
Source: Ecol Evol. 2014 Sep 30;4(20):3978–90. doi: 10.1002/ece3.1254 (PMC4242580; doi:10.1002/ece3.1254)
Supplement: Supplementary file 9 — Data S1. Optimality model of density-dependent resource use. [file ece30004-3978-SD9.docx]

**Supporting Information I**

**Optimality model of density-dependent resource use**

Here, we present an optimality model that predicts density-dependent niche expansion from ancestral wheat onto corn. The model is tailored to our experimental system (*Tribolium* flour beetles). However, since we consider a range of biologically realistic behaviors and mechanisms, the model should be generally applicable to any organism. We empirically parameterized variables in the model, to generate empirically based predictions as to how the optimal diet should change with density.

To generate a predictive model of niche shift in *Tribolium*, we constructed an optimality model of a population competing in a simple two-resource environment. We assume the consumer is better adapted to one resource (wheat, in the *Tribolium* system) and is exposed to a novel and sub-optimal alternative resource (corn). This situation is common when populations diversify to use newly available resources, such as host shifts in phytophagous insects. Initially, we use a frequency- and density-independent model of the optimal diet, then relax various assumptions and add further complications based on experimental data.

We assume population mean fitness, $\bar{w}$, depends on the fitness gained from each resource (*r_i_*, where *i* represents the ancestral resource *a* or novel resource *n*), weighted by the population’s proportional use of the novel resource ($D\in[0,1]$):

$\bar{w}$ *= (1 - D)r_a_ + Dr_n_* [**1**]

Note that writing $\bar{w}$ as a linear function of *D* ignores interactions between resources (e.g., nutritional complementarity); we relax this assumption later. Given this model relating fitness to diet, fitness is maximized either by *D* = 0 (if *r_a_* > *r_n_*) or *D* = 1 (if *r_a_* < *r_n_*), so the consumer should specialize on only one resource.

To introduce density-dependence, we assume that the fitness benefit *r_i_* of each resource is reduced by intraspecific competition as population density *N* increases:

*r_a_ = r_a_'(1 - α_a_N(1 - D))* [**2**]

*r_n_ = r_n_'(1 - α_n_ND)* [**3**]

where *r_i_'* is the density independent fitness on resource *i* (the intercept of the relationship between density and fitness on a given resource), and *α_i_* is the intrapopulation competition coefficient for resource *i*. Substituting these in Eq. **1**, we can calculate the optimal diet mix $\hat{D}$ at which (or for monotonic fitness functions, $\hat{D}$= 0 or 1). We can then evaluate how the optimal ratio of resources depends on population density, to predict the relationship between intraspecific competition and niche expansion. At low density, fitness is maximized when individuals specialize exclusively on the ancestral resource ($\hat{D}$ = 0, assuming *r_a_* > *r_n_*). However, as density increases, the cost of competition for the ancestral resource overwhelms its benefit and individuals should shift their diet onto the novel suboptimal resource to escape competition. The model predicts there is a threshold population density above which increased competition leads to greater reliance on the novel resource. Our model is a specific instance of a more general model known as the ideal free distribution, which suggests that individuals distribute themselves among resources in a manner that maximizes mean and reduces variance in population fitness (Fretwell and Lucas 1969; Morris 1987).

Note that our model does not track individual diet choice and thus omits among-individual diet variation. While such variation can alter model outcomes (e.g., via Jensen’s Inequality (Bolnick et al. 2011)), experimentally parameterizing and testing a more complete model would require that we estimate the variances and covariances within and among all parameters in the model, which is empirically prohibitive. We therefore focus on population mean fitness and mean resource use, while noting that population-level diet shifts can occur either due to changes in individual niche width and/or among-individual variation.

To obtain quantitative model predictions, we empirically parameterized free variables in Eqs. **1-3**, using the data from Experiment 1 (Fig. 1A, main text). We modified the model to reflect a log-linear relationship between fitness and density (Fig. 1A, main text):

$\bar{w}$ *= (1 – D) exp[r_a_’(1 – α_a_n(1 – D))] + D exp[r_n_’(1 – α_n_nD)]* [**4**]

to reflect the observation that competition is linearly related to the log mean fitness, rather than linearly related to mean fitness. The slope of the linear relationship between *r_i_* and the log of density (*N*) corresponds to *r_i_'α_i_* in Eqs. **2&3**, while *r_i_'* was estimated by the intercept of these lines. These parameters were estimated separately for each resource (Fig. 1A, main text).

By inserting these empirical values of *r_i_'*, and *α_i_* into Eq. **4** we predict optimal resource consumption $\hat{D}$ (proportion of novel resource) as a function of population density, when beetles are simultaneously offered both resources in adjoining patches (“WC”). At *N* = 1, the model predicts that fitness in WC is maximized when specializing on the ancestral resource (Supp. Info I Fig. 1A). Above a threshold density at *N* = 50, $\hat{D}$ begins to increase non-linearly with density (Supp. Info. I Fig. 1B), consistent with prior models suggesting that intraspecific competition should drive niche expansion.

Although our model assumes additive fitness effects of alternative resources (Eqs. **1&4**), previous work documents multiple examples of synergy between nutrient components of alternative diets ((Bjorndal 1991; Pennings et al. 1993). Departures from additive fitness may arise if resources contain complementary nutrients, or if a mixed diet allows higher total resource consumption or more efficient digestion. Due to the assumed additive effect, our basic model predicts that at low density beetles should specialize exclusively on wheat (because r*_a_* > *r_n_*). However, even at low density (*N* = 20), the WC experimental beetles consumed a moderate proportion of corn (*D* ~ 0.2; Fig. 3A, main text), suggesting that fitness may not be a linear function of *D.* The non-linear effect of diet on fitness was confirmed by experimentally manipulating diet and observing the resulting fitness (Experiment 2; Fig. 2, main text). Individual beetles’ fitness is maximized by a mixed diet with ~41% corn, explaining why they consumed a mixed diet even at low density.

To incorporate the fitness benefit of a mixed diet, we introduced a term in the model representing increased fitness proportional to the variance in resource use, *D*(1-*D*):

$\bar{w}$ *= (1 - D) exp[r_a_'(1 - α_a_(N- 1)(1 - D))] + D exp[r_n_'D(1 - α_n_(N- 1))] + D(1 - D)r_an_* [**5**]

We estimated the value of *r_an_* (the quadratic coefficient in Fig. 2 main text) and recalculated the optimal diet as a function of density. The revised model correctly predicts non-zero values of $\hat{D}$ at low densities, but still predicts density-dependent niche expansion onto corn. Thus, synergy between resources resolves one data-theory contradiction, but cannot explain the niche contraction. As described in the main text, this synergy is also observed at higher population density (Fig. 2B). Thus, a density-dependent loss of diet synergy cannot explain the observed niche contraction at higher densities.

We next incorporate the possibilities of trade-offs between fitness on the two resources. Trade-offs can arise in two ways. First, fitness on wheat and corn could be negatively correlated across genotypes (i.e. wheat-adapted genotypes are less fit on corn and vice versa). This is not supported by existing data: fitness on wheat and corn are not correlated among genotypes (Agashe et al. 2011). Alternatively, individuals might perform worse on one resource after exposure to a second resource (e.g., Persson 1985; Lewis 1986; Ackermann and Doebeli 2004; Agashe and Bolnick 2010). At first glance, such a tradeoff seems incompatible with our experimental finding that mixed-diet strategies maximize fitness (Fig. 2). However, these experiments do not address the tradeoff hypothesis proposed here because beetles either did not face any competition (Fig. 2A) or competed against individuals with the same mixed diet (Fig. 2B), but were not subject to competition with individuals of a different diet (this would be difficult to engineer with this experimental system). Foraging trade-offs are well known in many organisms. For instance, some *Enhydra lutris* individuals (California sea otters) specialize on a narrow set of prey taxa, and others are generalists (Sokoloff 1977; Estes et al. 2003; Agashe and Bolnick 2010). The generalists require much longer handling times on any single prey item (Via 1999; Tinker et al. 2008), which would tend to generate the types of competitive trade-offs we propose. Similarly, perch trained to recognize one prey item over a span of a few weeks rapidly lose this acquired search image after using another prey item (Persson 1985; Agashe and Bolnick 2012). This effect is likely to generate selection against generalist strategies with poor search ability on all prey.

We find that a model accounting for such trade-offs predicts density-dependent niche contraction under certain circumstances. We consider the possibility that each additional unit of corn in the diet reduces competitive ability for wheat (and vice versa). The reduction *a_i_* in fitness due to an additional competitor on resource *i* could be exacerbated at rate *γ_j_* with each additional unit of the other resource *j* in the diet (Supp. Info. I Fig. 2):

*α_a_ = α_a_'(1 + γ_a_D)* [**6**]

*α_n_ = α_n_'(1 + γ_n_(1 - D))* [**7**]

For example, specialists on wheat suffer a per-capita decline *α_w_'* for each additional competitor who uses wheat. On the other hand, individuals who consume 90% corn have their wheat-derived fitness penalized by *α_w_'*(1+0.9*γ_w_*) for each additional competitor on wheat. Therefore, with increasing consumption of the novel resource (increasing *D*), the competition coefficient on the ancestral resource (*α_a_*) increases so that individuals are increasingly poor competitors for the ancestral resource. Likewise, with decreasing *D* (and therefore increasing consumption of the ancestral resource), the coefficient of competition on the novel resource (*α_n_*) increases, which implies that individuals are increasingly ineffective competitors for the novel resource. Replacing Eqs. **6**&**7** into Eq. **4**, we get:

$\bar{w}$ *= (1 – D) exp[r_a_'(1 - [α_a_'(1 + γ_a_D)]N(1 - D))] + D exp[r_n_'(1 - [α_n_'(1 + γ_n_(1 - D))]ND)]* [**8**]

Unfortunately, estimating *γ_j_* is not empirically feasible in this system: it would require that we measure the fecundity of individuals who are fed a fixed mixture of wheat:corn, during competition with conspecifics on a single resource. Because beetles both live in and consume the flour they are provided, there is no way to feed individual beetles a specific diet mix while measuring their competitive ability on a homogenous resource. Lacking empirical estimates of *γ_j_*, we thus considered a range of values of *γ_a_* and *γ_n_* in Eq. **8**, combined with empirical estimates of all other parameters (above).

We again calculated the optimal proportion of corn as a function of population density, for various values of *γ_j_*. We found that *γ_a_* has a strong density-dependent effect on the relationship between niche width (increasing with % corn, *D*) and fitness (Supp. Info. I Fig. 2). When *γ_a_* is equal to 0, there are no trade-offs, and the model predicts density-dependent niche expansion as described above (Fig. 3, main text). However, the fitness function changes drastically at *γ_a_* > 1.5. At *γ_a_* = 1.5, the competition coefficient for wheat is 75% higher for generalists (at *D* = 0.5) than for wheat specialists (at *D* = 0). This trade-off is only important when density is high enough that the competition coefficient appreciably reduces fitness. Thus, as density increases, generalists are penalized when in competition with specialists (which was not the case Supp. Info. I Fig. 1). As a result, our model predicts density-dependent niche expansion when *γ_a_* < 1.5, and no change in niche breadth when *γ_a_* > 1.5 (Supp. Info. I Fig. 2D). Interestingly, we find that the optimal diet composition is largely unaffected by the cost of generalization for competitive ability on corn. The negligible effect of *γ_n_* can be explained by the comparatively small contribution of corn flour to the overall fitness of the beetle population, and correspondingly weaker competition on corn. More importantly, density-dependent niche expansion reappears at all values of *γ_a_* (including *γ_a_* > 1.5) when we add the synergistic effect of combining the two resources as proposed in Eq. **5** (Supp. Info. I Fig. 3). Our model consistently predicted niche expansion under the 3-fold range of values for *γ_a_* we considered. In principle, very strong trade-offs could overwhelm the benefits of diet synergy, but such strong trade-offs would be inconsistent with our empirical observation that generalist individuals consuming both wheat and corn have higher fitness, at both low and high density.

**
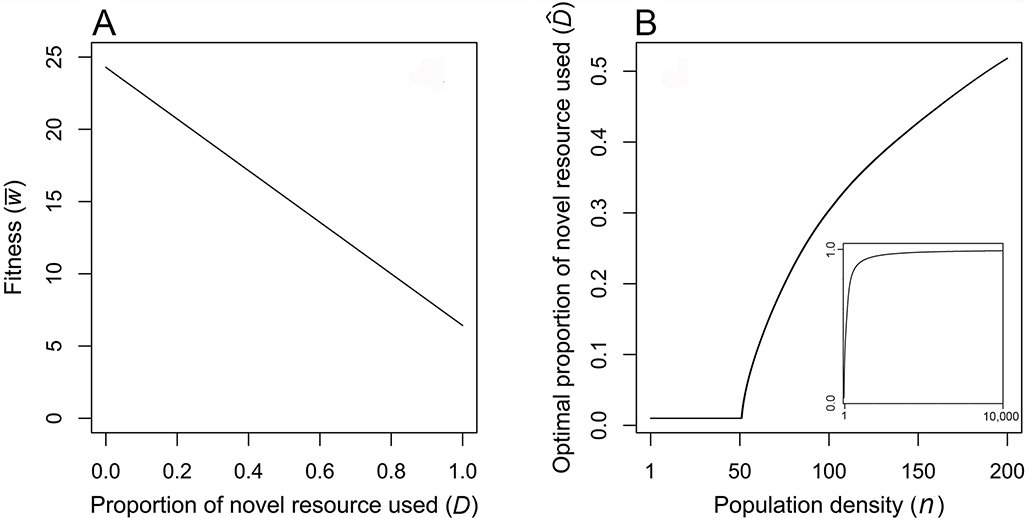
**

**Supp. Info. I Figure 1.** Predictions from the paramaterized basic model of density-dependent resource use. (A) Predicted density-independent fitness as a function of the proportion of novel resource consumed. (B) Predicted density-dependent optimal proportion of novel resource, for *N* = 1 to 200, and *N* = 1 to 10,000 in the inset. Parameter values: *r_a_'α_a_* = -0.0107; *r_a_'* = 3.19*; r_n_'α_n_* = -0.0129; *r_n_' =*1.86 (see Fig. 1A of main text).

**
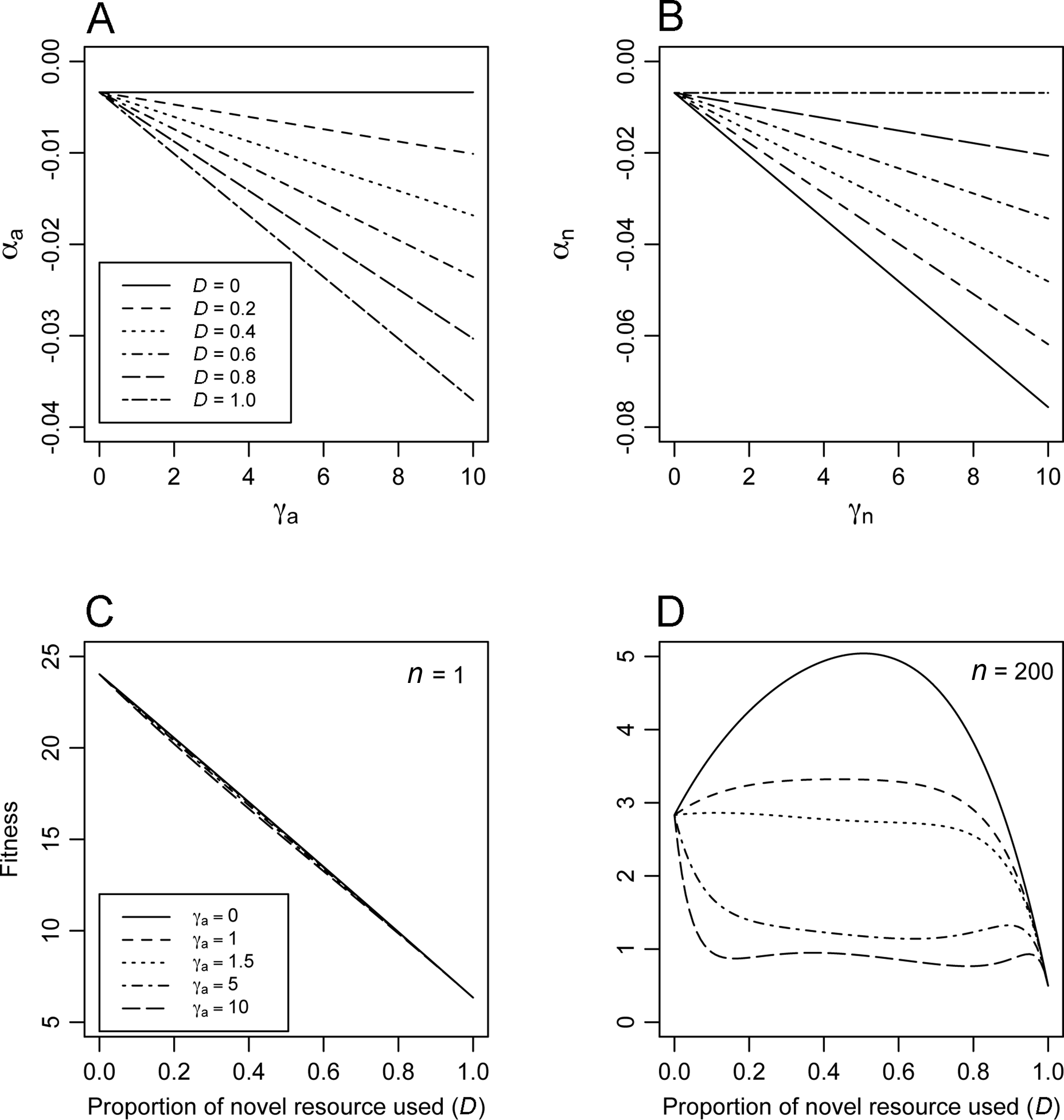
**

**Supp. Info. I Figure 2.** (A and B) Effect of *γ_j_* on *a_i_*, where *a_i_* = the intrapopulation competition coefficient for resource *i*, and *γ_j_* = the rate at which *a_i_* increases with each additional unit of the other resource *j* in the diet. The lines represent different proportion of the novel resource included in the diet (*D*). (C and D) Effect of *γ_j_* on the relationship between *D* and population fitness ($\bar{w}$), at low (C) and high (D) population density. The lines represent different values of *γ_a_*. (*a* and *n* correspond to the ancestral and novel resource, respectively).
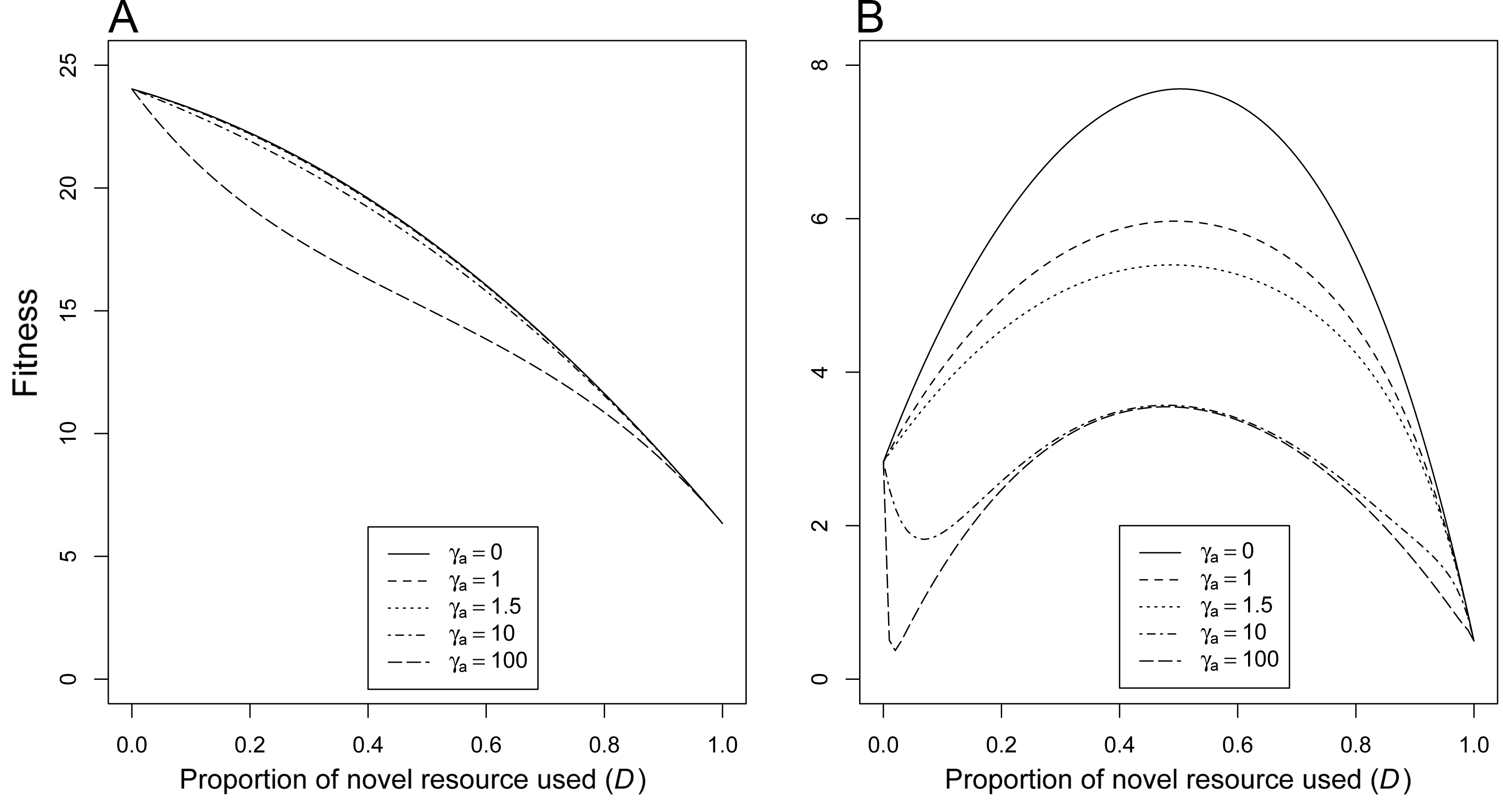


**Supp. Info. I Figure 3.** Effect of different values of *γ_j_* on the relationship between *D* and population fitness ($\bar{w}$) at (A) low (*N* = 1) and (B) high (*N* = 200) population density, in a model that includes the synergistic effect of combined resources (as observed in Fig. 2, main text).

**Literature cited in Supporting information I**

Ackermann, M., and M. Doebeli. 2004. Evolution of niche width and adaptive diversification. Evolution 58:2599–2612.

Agashe, D., and D. I. Bolnick. 2012. Dietary niche and population dynamic feedbacks in a novel habitat. Oikos 121:347–356.

Agashe, D., and D. I. Bolnick. 2010. Intraspecific genetic variation and competition interact to influence niche expansion. Proc Biol Sci 277:2915–2924.

Agashe, D., J. J. Falk, and D. I. Bolnick. 2011. Effects of founding genetic variation on adaptation to a novel resource. Evolution 65:2481–2491.

Bjorndal, K. A. 1991. Diet mixing: nonadditive interactions of diet items in an omnivorous freshwater turtle. Ecology 72:1234.

Bolnick, D. I., P. Amarasekare, M. S. Araujo, R. Buerger, J. M. Levine, M. Novak, V. H. W. Rudolf, S. J. Schreiber, M. C. Urban, and D. A. Vasseur. 2011. Why intraspecific trait variation matters in community ecology. Trends Ecol Evol 26:183–192.

Estes, J., M. Riedman, M. Staedler, M. Tinker, and B. Lyon. 2003. Individual variation in prey selection by sea otters: patterns, causes and implications. J Anim Ecol 72:144–155.

Fretwell, S. D., and H. L. Lucas Jr. 1969. On territorial behavior and other factors influencing habitat distribution in birds. Acta Biotheor 19:16–36.

Lewis, A. 1986. Memory constraints and flower choice in *Pieris rapae*. Science 232:863–865.

Morris, D. 1987. Spatial scale and the cost of density-dependent habitat selection. Evol Ecol 1:379–388.

Pennings, S. C., M. T. Nadeau, and V. J. Paul. 1993. Selectivity and growth of the generalist herbivore *Dolabella auricularia* feeding upon complementary resources. Ecology 74:879.

Persson, L. 1985. Optimal foraging: the difficulty of exploiting different feeding strategies simultaneously. Oecologia 67:338–341.

Sokoloff, A. 1977. The Biology of *Tribolium* With Special Emphasis on Genetic Aspects. Oxford University Press, London.

Tinker, M. T., G. Bentall, and J. A. Estes. 2008. Food limitation leads to behavioral diversification and dietary specialization in sea otters. PNAS 105:560–565.

Via, S. 1999. Cannibalism facilitates the use of a novel environment in the flour beetle, *Tribolium castaneum*. Heredity 82:267–275.
