# Supplementary material for: Intraspecific competition reduces niche width in experimental populations
Source: Ecol Evol. 2014 Sep 30;4(20):3978–90. doi: 10.1002/ece3.1254 (PMC4242580; doi:10.1002/ece3.1254)
Supplement: Supplementary file 10 — Data S2. Linear programming models of optimal foraging theory. [file ece30004-3978-SD10.docx]

**Supporting Information II**

In this appendix, we show that linear programming models of optimal foraging theory are capable of predicting density-dependent niche contraction, given certain assumptions. This makes the point that competition does not necessarily drive niche expansion, in contrast to the optimality model outlined in Supporting Information I. We focus on a strictly graphical model that is sufficient to make the point that competition can drive niche contraction.

We begin by assuming that a consumer relies on some mixture of two resources (e.g., wheat and corn) and that time spent foraging for or consuming one resource cannot be spent foraging for or consuming the other resource. Because time spent foraging for each resource (t_w_, t_c_) and on other activities (t_o_) cannot exceed total available time within a day (T > t_w_ + t_c_ + t_o_), there are constraints on total time spent foraging (Fig. SII.A). Rearranged, total foraging time cannot exceed T – t_o._

On top of the time constraints, we add constraints to digestive capacity. The total amount of wheat consumed (C_w_) depends on the time spent foraging for wheat, t_w_, times the abundance of wheat per unit foraging time, D_w_, such that C_w_ = t_w_D_w_, and similarly C_c_ = t_c_D_c_. Neither value can increase without bounds, due to constraints of gut volume and digestive rate, so that the maximum total amount of wheat consumed per day is capped at some maximum gut volume V_w_ which may or may not equal the maximum amount of corn consumed per day, V_c_. That is, V_w_ ≥ t_w_D_w_ and V_c_ ≥ t_c_D_c_, but V_w_ and V_c_ may not be equal because (for instance) one may be digested more slowly than the other. Finally, we add in the likelihood that total stomach volume is constrained regardless of the ratio of food consumed, such that V_w_ + V_c_ ≤ V_max_. This generates a second constraint line in Fig. SII.B that represents the upper boundary of food intake set by gut volumes.

If, as shown in Fig. SII.B, one resource fills the gut more quickly than the other, then the gut fullness constraint and the time constraint lines may intersect, though this does not always have to be true. When the two constraint lines intersect, there will be some range of resource ratios in which total energy income is limited mostly by time constraints, and other resource ratios where income is limited by stomach volume constraints. More importantly, there will be some point (green dot in Fig. SII.C) denoting a combination of resources (arrows in Fig SII.C) that maximize food intake. This point represents an optimal foraging strategy in the face of the simultaneous constraints on stomach volume and foraging rates. At the point illustrated in Fig. SII.C, increased wheat consumption is constrained by time, and increased corn consumption is constrained by stomach volume.

If the optimal foraging strategy entails some mixture of resources as shown in Fig. SII.C, then competition can drive niche contraction as follows. First, we assume that competition degrades either forager physiological performance or resource quality, such that it takes longer to digest consumed resources. If this disproportionately affects one resource (e.g., corn), then competition alters the slope of the stomach volume constraint (red line in Figs. SIIB-D). That altered slope changes the intersection point between the time and digestive constraint lines, and thereby alters the optimal ratio of wheat and corn consumption (while also reducing total resource intake). In the example illustrated in Fig. SII.D, resource competition may slow the rate of digestion of corn, reducing the maximal volume of corn consumed per day (V_c_ is reduced), shifting the area of allowable diets and the optimum to the new point denoted in Fig. SII.D. At this new optimum, the population uses predominantly wheat and very little corn, representing niche contraction in response to density.

We emphasize two key points regarding this model. First and most importantly, the density-dependent niche contraction illustrated here is not the only possible outcome from linear programming. Different arrangements of line slopes, constraints, or different assumptions about how competition modifies time and digestive constraints can lead to different outcomes. For example one might assume instead that competition leads to interference among individuals, increasing time spent fighting and reducing total time available for foraging. This shifts the blue line in Figs. SII closer towards the origin (reducing T-t_o_ because t_o_ increases), and changes the optimum diet. Depending on the arrangement of other (digestive constraint) intercepts, reduction in foraging time could lead to more use of corn (as illustrated in Fig. SII.E), or more use of wheat (not shown, requires a diff erent arrangements of the two constraint lines).

A second point is that this model still relies on optimality. That is, although total energy income may go down when competition reduces foraging time or digestive ability, individuals are still assumed to choose foraging efforts that maximize their fitness (green points in Figs. SIIB-E). In contrast, our empirical results show that individuals are deviating from the optimum diet ratio and so do not appear to match this, or any other, optimality model.


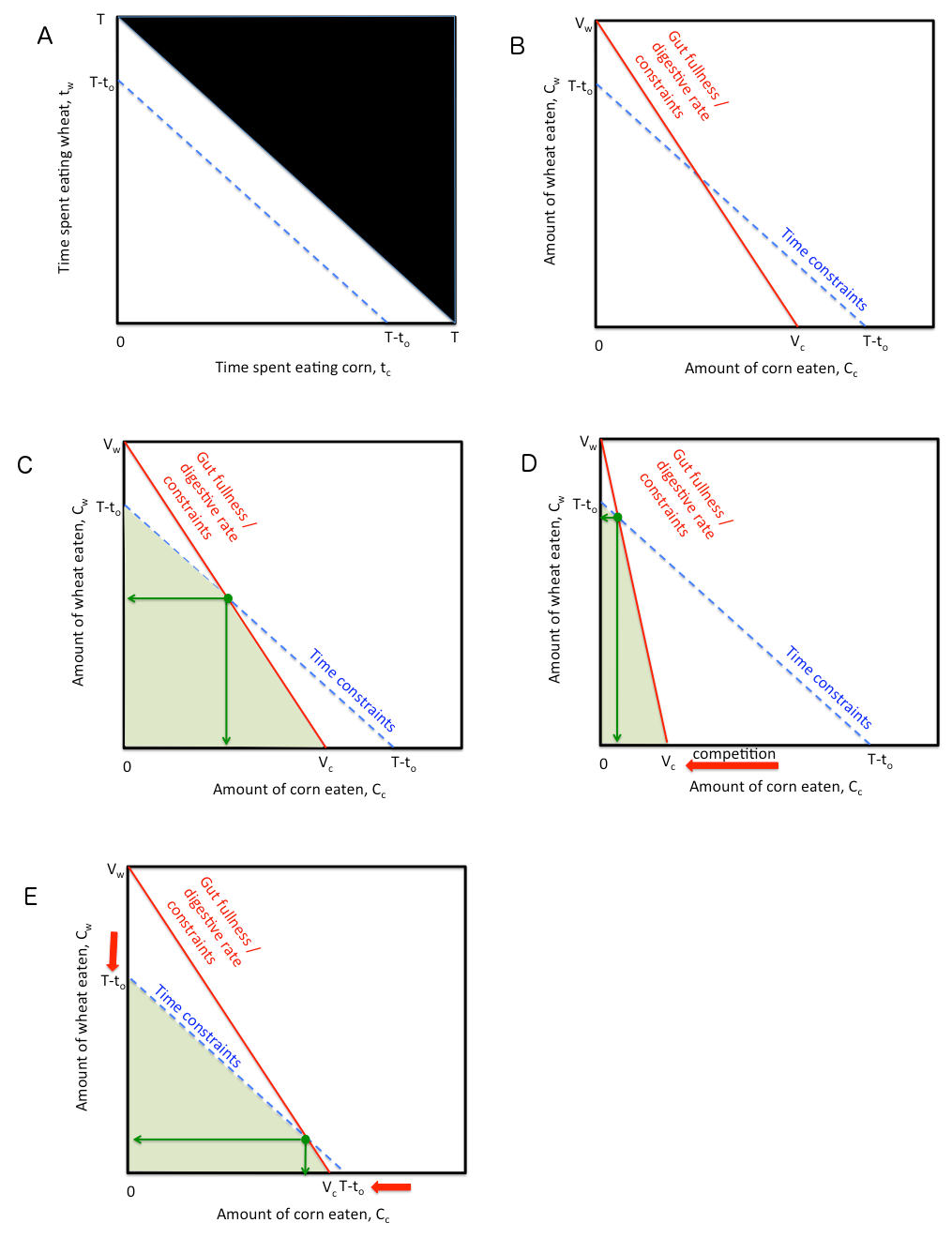


Figure SII. A graphical illustration of a linear programming model of optimal foraging under simultaneous foraging time and digestive constraints for foragers faced with two alternate resources (wheat versus corn, for example, to match our empirical system). (A) illustrates foraging time constraints alone, with the black shaded area being disallowed combinations of foraging times that exceed total time available, and the dashed blue line indicating the upper boundary of foraging time combinations on either of two resources (e.g., wheat versus corn) after accounting for other time demands. A second constraint due to digestive capacity is added in panel (B), as described in the Supplement II text and shown here by a red solid line. Panel (C) is the same as (B) except that we now shade in green the area of allowed foraging efforts on two resources. The maximal foraging effort is the upper/right boundary to this polygon, and the optimum (assuming equal energetic value of the two resources and additive/substitutive nutritional effects) is the point along the boundary that maximizes total intake (indicated by a green dot). This optimum corresponds to a rate of foraging on wheat and corn that is optimal in the sense that it maximizes energy intake, subject to the constraints indicated. Competition can modify these constraints either by changing digestive capacity (D) or foraging time (E), resulting in shifts of the optimal foraging combination.
